# Revision of *Mandarella* Duvivier from Taiwan, with a new species, new synonymies and identities of highly variable species (Insecta, Chrysomelidae, Galerucinae, Alticini)

**DOI:** 10.3897/zookeys.568.7125

**Published:** 2016-02-23

**Authors:** Chi-Feng Lee, Cheng-Lung Tsai, Alexander Konstantinov, Wen-Bin Yeh

**Affiliations:** 1 Applied Zoology Division, Taiwan Agricultural Research Institute, 189 Chung-Cheng Road, Wufeng, Taichung 41362, TAIWAN; 2 Department of Entomology, National Chung Hsing University, 250 Kuo Kuang Road, Taichung 40227, TAIWAN; 3 Systematic Entomology Laboratory, MRC-168 Washington, USA

**Keywords:** Flea beetles, alpine, molecular, taxonomic revision

## Abstract

Taiwanese species of *Mandarella* Duvivier are compared on the basis of morphological and molecular evidence. Only three of eleven morphospecies are considered to be valid. *Mandarella uenoi* (Kimoto, 1969) is transferred from the genus *Luperus* Geoffroy. *Stenoluperus taiwanus* Kimoto, 1991 and *Stenoluperus kimotoi* Döberl, 2001 are synonymized with *Mandarella uenoi*. Taiwanese records of *Stenoluperus tibialis* Chen, 1942, *Stenoluperus nipponensis* Laboissière, 1913, and *Stenoluperus potanini* (Weise, 1889) were based on misidentifications and represent *Mandarella uenoi*. The Taiwanese population previously erroneously identified as *Stenoluperus pallipes* Gressitt and Kimoto, 1963 is here described as a new species, *Mandarella tsoui* **sp. n.**, *Stenoluperus esakii* Kimoto, 1969, *Stenoluperus matsumurai* Takizawa, 1978, and *Mandarella taiwanensis* Medvedev, 2012 are synonymized with *Mandarella flaviventris* (Chen, 1942).

## Introduction

*Mandarella* Duvivier, 1892 is a small genus of flea beetles containing five species (Döberl 2010). *Stenoluperus* Ogloblin, 1936 was a larger genus (33 species) and was proposed as a junior synonym of *Mandarella* Duvivier by Medvedev (2012). This synonym is confirmed after/by examination of type species of both genera (Konstantinov, personal communication). Thus, *Mandarella* now contains 34 valid species limited to the Palaearctic region.

**Figures 1–8.**
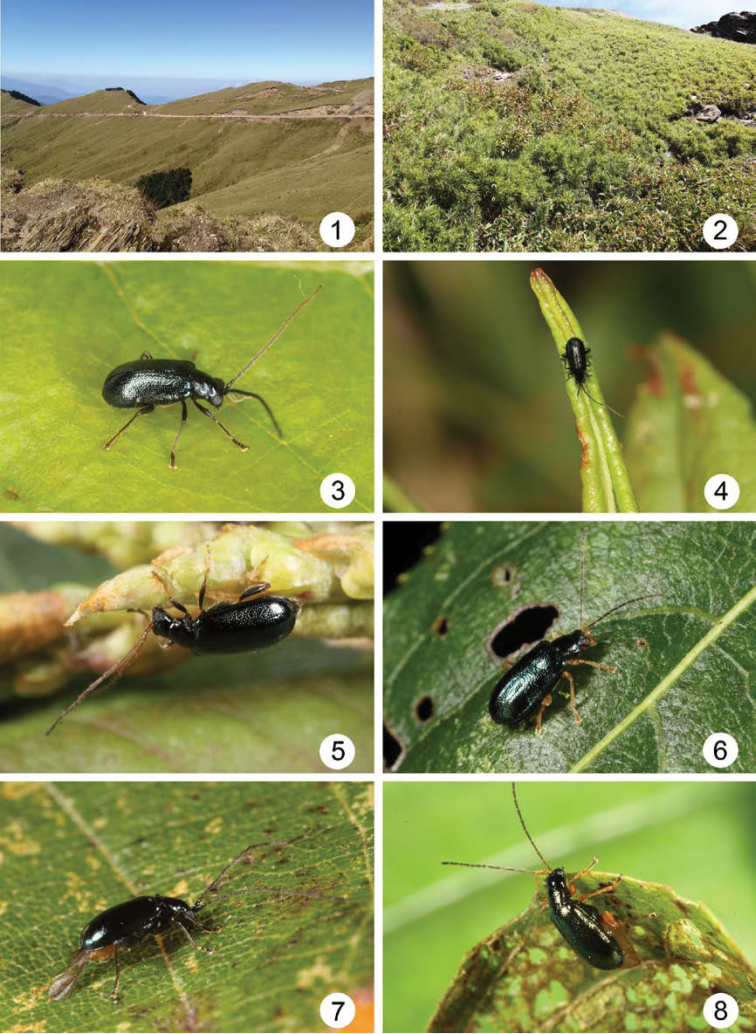
Field photographs. **1** Alpine habitat, Hohuanshan **2** Microhabitat **3** *Mandarella uenoi* form C **4** *Mandarella uenoi* form B **5** *Mandarella uenoi* form D **6** *Mandarella tsoui* sp. n. **7** *Mandarella flaviventris* form G **8** *Mandarella flaviventris* form I.

More than 250 mountains exceed 3000 meters in Taiwan. Localities higher than 3000 m present cold and windy montane habitats (Figs 1–2) where few insects can survive, including leaf beetles. However, members of *Mandarella* are adapted to these habitats and display extensive morphological diversity (Figs 3–8). Based on morphological characteristics of body color and antennomeres, 11 species, mostly from limited montane areas, have been reported from Taiwan. Chûjô (1965) recorded the first two species, *Stenoluperus flaviventris* Chen, 1942 and *Stenoluperus tibialis* Chen, 1942. Kimoto (1969) described *Stenoluperus esakii*, reported *Stenoluperus pallipes* Gressitt and Kimoto, 1963 and *Stenoluperus nipponensis* Laboissière, 1913. Kimoto (1974) subsequently recorded *Stenoluperus potanini* (Weise, 1889). Takizawa (1978) described *Stenoluperus matsumurai*. Kimoto (1991a) described *Stenoluperus taiwanus*. At the same year, Kimoto (1991b) described *Stenoluperus minor* and *Stenoluperus itoi*. However, *Stenoluperus minor* Kimoto 1991b was a primary junior homonym of *Stenoluperus minor* Kimoto, 1977 and was replaced as *Stenoluperus kimotoi* by Döberl (2001). *Stenopluperus itoi* Kimoto, 1991b was a secondary junior homonym of *Mandarella itoi* Chûjô, 1966 and was replaced as *Mandarella taiwanensis* by Medvedev (2012). We have discovered that *Luperus uenoi* Kimoto, 1969 is also a member of *Madarella* based on examination of the type specimens.

Although these *Mandarella* species can be separated by color patterns and relative lengths of antennomeres (Kimoto and Takizawa 1997), only three forms of male aedeagi were found during our study. In addition, previous authors have noted that variations in body color and antennomeres may be the result of local adaptation that is not indicative of species boundaries (Kurachi et al. 2002, Nahrung and Allen 2005, Quinzin and Mardulyn 2014). Molecular approaches have been applied broadly in systematics of various insects and have fueled taxonomic debates about species recognition, especially when morphological characters are insufficient (Brown et al. 2012, Kumar et al. 2012; Lee et al. 2013, Lees et al. 2014, Park et al. 2011, Tsai et al. 2014). Sequences data from cytochrome oxidase subunit I (COI), a small fragment of mitochondrial DNA, have been viewed as efficient markers in Chrysomelidae and have been exploited to resolve debates in identification (Germain et al. 2013, Kubisz et al. 2012, Lopes et al. 2015) and elucidate species complex phylogenetics (Quinzin and Mardulyn 2014). Nie et al. (2012) also used COI to clarify two closely related leaf beetles with color variation in elytra and pronota. In the present study, mtDNA COI markers are used to examine the taxonomy of 11 species of *Mandarella* leaf beetles that vary locally in morphology and color.

## Methods

### Depositories of material examined

BPBM
 Bernice P. Bishop Museum, Hawaii, USA [James Boone] 


CAS
California Academy of Sciences, California, USA [David H. Kavanaugh] 


EIHU
 Systematic Entomology, The Hokkaido University Museum, Sapporo, Japan [Masahiro Ôhara] 


EUMJ
 Entomological Laboratory, Faculty of Agriculture, Ehime University, Matsuyama, Japan [Hiroyuki Yoshitomi] 


KMNH
 Kitakyushu Museum of Natural History and Human History, Kitakyushu, Japan [Yûsuke Minoshima] 


KUEC
Faculty of Agriculture, Kyushu University, Fukuoka, Japan [Osamu Tadauchi] 


NMNS
National Museum of Natural Science, Taichung, Taiwan [Ming-Luen Jeng] 


TARI
Taiwan Agricultural Research Institute, Taichung, Taiwan 


Exact label data are cited for type specimens and voucher ones of the described species; a double slash (//) divides the data on different labels and a single slash (/) divides the data in different rows. Other comments and remarks are in square brackets: [p] – preceding data are printed, [h] – preceding data are handwritten, [w] – white label, [y] – yellow label, [b] – blue label, [r] – red label, and [y] – yellow label.

### Specimens and sampling

Approximately 2500 specimens were examined for this study. Most of them either belong to the historic collections at TARI or were collected recently as part of a long term project “inventorying chrysomelids of Taiwan” by the Taiwan Chrysomelid Research Team (TCRT). One hundred and thirty-five specimens were collected for DNA analysis. These specimens were classified into morphospecies, including two specimens of *Mandarella nipponensis* collected from Japan. *Dercetina itoi* Kimoto, 1969 and *Dercetina shirozui* Kimoto, 1969 were used as outgroups for convenience since they fed on the same host plants as *Mandarella tsoui* sp. n.

### DNA extraction, amplification, and sequencing

Genomic DNA was extracted from the meta-femora via QuickExtract DNA extraction kits (Epicenter Biotechnologies, Madison, WI). The protocol was modified according to Tsai et al. (2014). The primer sets used to amplify the mitochondrial COI gene are listed (Table 1). Polymerase Chain Reaction was conducted in a volume of 25 μl and the programing conditions were 94 °C for 2 min for initial denaturation, 35 cycles of 94 °C for 40 s, 45 °C for 40 s, and 72 °C for 40 s, then 72 °C for 10 min as a final extension. The upstream primer COI-49_Chrysomelidae_F and COI-64_Chrysomelidae_F were used for COI if COI-Chry_F was not successful in achieving amplification. Purification of PCR products was conducted via QIA quick Gel Extraction Kit (Qiagen, Hilden, Germany) from 1% agarose gel. DNA products were sequenced using Taq dye terminator Cycle Sequencing Kit (Applied Biosystems, Foster City, CA) and an ABI 377A sequencer.

**Table 1.** Primers and their amplification size in this study.

Gene	Primer	Sequence 5’→3’	Size (bp)	References
COI	COI-Chry_F (+)	ACYAAYCAYAAAGAYATWGG	689	In this study
	COI-49_Chrysomelidae_F	CATAAAGATATTGGHACHTT	683	
	COI-64_Chrysomelidae_F	ACHYTRTAYTTYATTTTYGG	668	
	CI-731Coleoptera (-)	CCAAAAAATCAAAATAAATGTTG		Tsai et al. (2014)

“+” and “-” are upstream and downstream primers, respectively.

### Phylogenetic analyses

Sequences were edited in Bioedit 7.0 (Hall 1999) and then aligned using Muscle Multiple Alignment option in SeaView4 (Gouy et al. 2010). Genetic divergences among species were analyzed using MEGA 6.0 via p-distance (Tamura et al. 2013).

Phylogenetic inference of COI was conducted using neighbor-joining clustering (NJ) and Bayesian inference (BI). For NJ, Kimura two-parameter (K2P) was selected as the substitution model and 1000 replications of bootstrapping analyses were applied. For BI, the evolutionary hypothesis of nucleotide substitution inferred in jModelTest 0.1 (Posada 2008) using Bayesian Information Criterion (BIC) for the best-fit models of COI was TIM3 + I + G. BI of the COI gene was analyzed in MrBayes v. 3.2 (Ronquist et al. 2012) with three heat chains and one cold chain, and MCMCMC searches were conducted for 1 × 10^7^ generations with sampling every 100 generations. Analysis was finished with the average standard deviation of split frequencies below 0.01. The initial 25% of trees were discarded as burn-in, and then the remaining trees were used to generate a consensus tree.

## Results

A total of 131 specimens of *Mandarella* flea beetles were successfully amplified for COI, with 584 bp in this study. The average nucleotide compositions for G, A, T, C are 18.4%, 28.0%, 32.8%, 20.8%, respectively. All sequences have been deposited in GenBank (Suppl. material 1).

**Figure 9.**
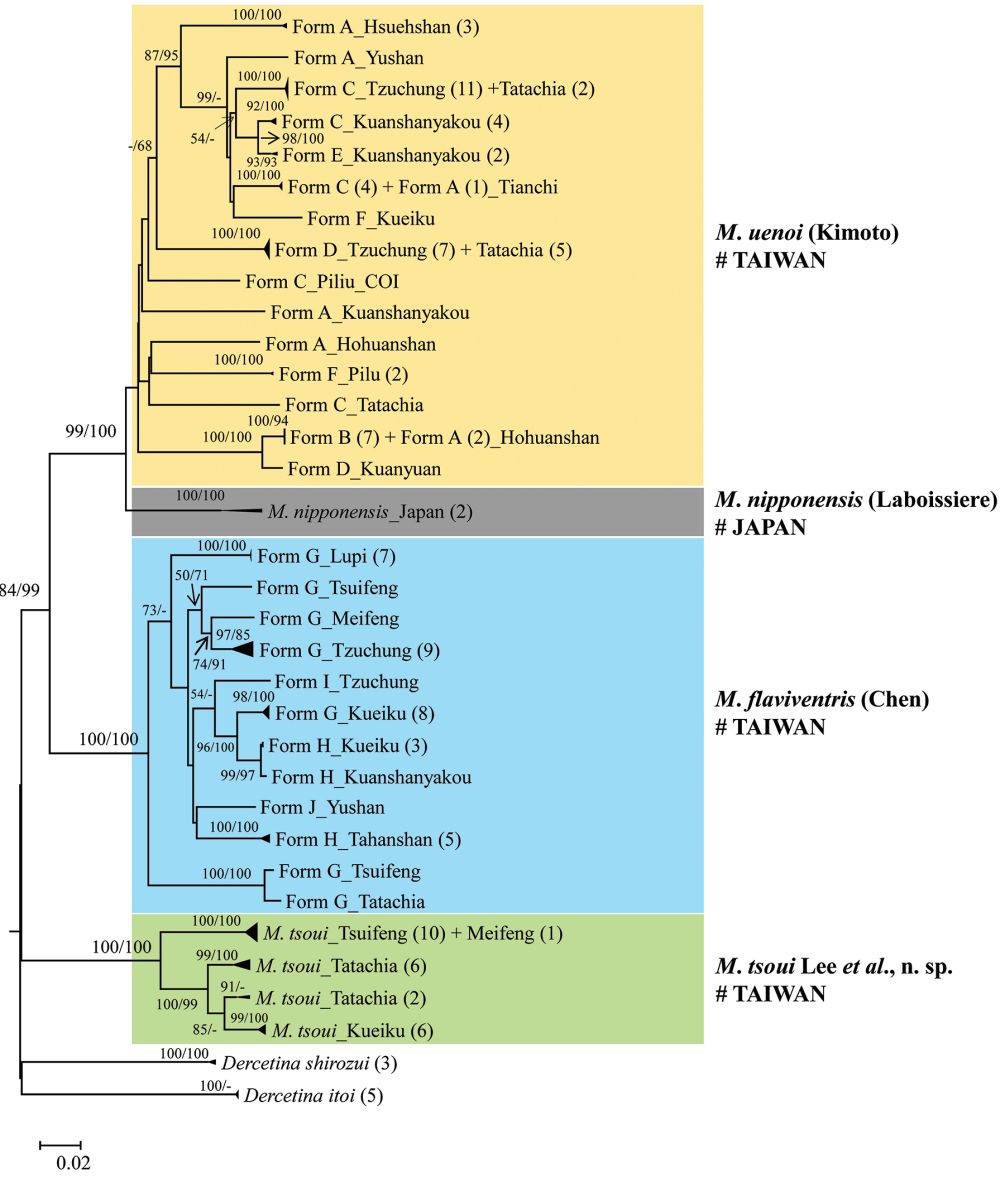
Neighbor-joining (NJ) inference based on COI gene using Kimura two-parameter (K2P) substitution model. Both bootstrapping values of NJ (left) and those of posterior probabilities from Bayesian inference (BI) (right) more than 50% are shown at nodes.

Phylogenetic inferences based on COI gene using neighbor-joining (NJ) and Bayesian inference (BI) reveal that montane *Mandarella* flea beetles are monophyletic, with three lineages, each including several morphospecies (Fig. 9). In the *Mandarella uenoi* lineage, Japanese populations of *Mandarella nipponensis* diverge separately, while the other morphology-based Taiwanese taxa (i.e., *Mandarella kimotoi*, *Mandarella nipponensis*, *Mandarella potanini*, *Mandarella taiwana*, *Mandarella tibialis*) collected from different montane areas overlap with each other. A similar situation exists in the *Mandarella flaviventris* lineage, with four morphospecies, *Mandarella flaviventris*, *Mandarella esakii*, *Mandarella matsumurai*, and *Mandarella taiwanensis*, from different localities overlapping and mixing. The third lineage includes several flea beetles in a separate group that occur only in mainland China. Therefore, specimens collected from Tsuifeng, Tatachia and Kueiku forming a separate lineage should be considered a new species, i.e. *Mandarella tsoui* sp. n.

Among *Mandarella* flea beetles, the interspecific genetic distances range from 16.2–22.6%, while the intraspecific divergence is 0.0–14.4%.

## Systematics

### Mandarella uenoi

Taxon classificationAnimaliaColeopteraChrysomelidae

(Kimoto, 1969) comb. n.

Stenoluperus tibialis : Chûjô 1965: 98 (Taiwan); Kimoto 1969: 40 (additional records in Taiwan); Kimoto 1991c: 13 (additional record in Taiwan). **Misidentification**
Mandarella tibialis : Medvedev 2012: 427.
Luperus uenoi Kimoto, 1969: 39 (Taiwan).
Stenoluperus nipponensis : Kimoto 1969: 40 (Taiwan); Kimoto 1989: 253 (additional records in Taiwan); Kimoto 1991c: 12 (additional records in Taiwan). **Misidentification**
Stenoluperus potanini : Kimoto 1974: 26 (Taiwan); Kimoto 1989: 253 (additional records in Taiwan); Kimoto 1991c: 12 (additional records in Taiwan). **Misidentification**
Stenoluperus taiwanus Kimoto, 1991a: 14. **New synonym**
Mandarella taiwana : Medvedev 2012: 427.
Stenoluperus minor Kimoto, 1991b: 117 (nec Kimoto, 1977).
Stenoluperus kimotoi Döberl, 2001: 383. (replacement name for Stenoluperus minor Kimoto, 1991). **New synonym**

#### Type material.

*Luperus uenoi*. Holotype ♂ (KUEC): “(Taiwan) / Mt. Nan-hu-ta Shan [南湖大山] / 3,580 m / Tái-chung Hsien [h, w] // 17.VI. [h] 19 [p] 61 [h] / S. Ueno [p, w] // *Luperus* / *uenoi* / Kimoto, sp. n. [h, w] // HOLOTYPE [p, r]”. Paratypes: 1♂, 1♀, (KMNH): “(Taiwan) / Mt. Nan-hu-ta Shan [南湖大山] / 3,580 m / Tái-chung Hsien [h, w] // 17.VI. [h] 19 [p] 61 [h] / S. Ueno [p, w] // *Luperus* / *uenoi* / Kimoto, sp. n. [h, w] // PARATOPOTYPE [p, b]”.

*Stenoluperus minor*. Paratype: 1♂ (KMNH): “Mt. YUSHAN [玉山] / TAIWAN / 8. VI. 1980 / N. ITO [p, y] // *Stenoluperus* / *minor* / Kimoto, sp. n. [h] / Det. S. Kimoto, 19 [p] 91 [h, w] // PARATOPOTYPE [p, b]”.

*Stenoluperus taiwanus*. Paratypes: 1♀ (KMNH): “(Taiwan) / Yuanfeng [鳶峰], 2800m / -- Kunyang [昆陽], 3100m / Nantou Hsien [p, w] // *Stenoluperus* / *taiwanus* / Kimoto, sp. n. [h] // Det. S. Kimoto, 19 [p] 91 [h, w] // *Stenoluperus* / *tibialis* / Chen ? [h] / Det. S. Kimoto, 19 [p] 75 [h, w] // PARATYPE [p, b] // Japan-U. S. / Co-op. Sci. / Programme [p, y] // 1.VI.1965 / T. Nakane [h, w]”; 1♂ (KMNH): “Mt. Ho Huan Shan, [合歡山] / (3200m) / M-Taiwan / 28.V.1989 / Col. K. Baba [p, w] // *Stenoluperus* / *taiwanus* / Kimoto, sp. n. [h] // Det. S. Kimoto, 19 [p] 91 [h, w] // PARATYPE [p, b]”; 1♀ (KMMH): “ M.t Wu Kon Shan, [五公山] / near Liu kuei, / S-Taiwan / 3.VI.1989 / Col. K. Baba [p, w] // *Stenoluperus* / *taiwanus* / Kimoto, sp. n. [h] // Det. S. Kimoto, 19 [p] 91 [h, w] // PARATOPOTYPE [p, b]”.

#### Voucher specimens.

*Stenoluperus nipponensis*. 1♂ (KMNH): “(Taiwan) / Mt. Nanhu-pei Shan [南湖北山] / 3,500 m / I-lan Hsien [h, w] // 17.VI. [h] 19 [p] 61[h] / S. Ueno [p, w]”; 1♂ (KMNH): “Mt. Hsüeh Shan [雪山] / 3,400~3,600 m / Tái-chung Hsien [h, w] // 22.VI. [h] 19 [p] 61 [h] / S. Ueno [p, w]” (it belong to form *uenoi*); 1♂ (KMNH): “(Taiwan) / Alishan [阿里山] / Yushankou [玉山口] / Chiai Hsien [p, w] // May [p] 26 [h] .1971 [p] / K. Kanmiya [p, w]”; 1♀ (KMNH): “Ya Kou, [啞口] / Alt. Ca. 2800m. / Kao Hsiung Hsien, / S-Taiwan / 1.VIII.1986 / Col. K. Baba [p, w]”; they were determined by Kimoto in 1968. 1♀ (EUMJ): “(TAIWAN) / Tsuifeng [翠峰] / Nantou Hsien / 28. VI, 1970 / Y. Hor [p, w]”; it was determined by Kimoto in 1991.

*Stenoluperus potanini*. 1♀ (CAS): “Szechuan, W. China / Omei Shan: Shin-kai / -sze, 1,500 M. Aug. / 9. 1940. L. Gressitt [p, w]”; 1♂ (BPBM): “near Mupin / 7000-1300 ft [p] / Jul.6-8, [h] ’29 [p, w] // Szechuan / CHINA / DCGraham [p, w] // US [p, w]”; 1♂ (BPBM): “nr Mupin / Jul.7.1929 / 12,300 ft. [p, w] // Szechuan / CHINA / DCGraham [p, w] // ILL [p, y] // N48 [h, w]”; 1♀ (BPBM): “Washan [p] / 7-26-25 [h] / Szechuan [p, w] // China / Alt [p] 11,000 ft [h, w] // DCGraham / collector [p, w] // US [p, w]”; 1♀ (BPBM): “Szechuan, W. China / Omei Shan: S. side. / 2,000-1,000 M. Aug. / 12. 1940. L. Gressitt [p, w] // N62 [h, w] // ILL [p, y]”; 1♀ (BPBM): “Szechuan, W. China / Nien-hwo-shih to / summit. Omei Shan / 2,000-3,060 M. Aug / 10. 1940. L. Gressitt [p, w]”; they was determined by Gressitt & Kimoto in 1962. 1♂ (KMNH): “Mt. HOHUAN [合歡山] / TAIWAN / 3.V.1982 / T. ITO [p, y]; 1♀ (KMNH): “Ho Huan Shan, [合歡山] / Alt. 3200m. / Nan Tow Hsien. / M-Taiwan / 6.VIII.1986 / Col. K. Baba [p, w]” (it should belong to form *uenoi*); both were determined by Kimoto in 1991. 1♂, 1♀ (KMNH): “(FORMOSA) / Lake Yenyanfu [鴛鴦湖] / Ilan Hsien / 29, IV 1982 / N. Ohbayashi leg. [p, w]”; both were determined by Kimoto in 1987.

*Stenoluperus tibialis*. 1♂ (CAS): “Szechuan, W. China / Omei Shan: below / Shin-kai-sze, alt. 1,400-1,000 M. Aug. / 17. 1940. L. Gressitt [p, w]”; 1♀ (BPBM): “Szechuan, W. China / Nien-hwo-shih to / summit. Omei Shan / 2,000-3,060 M. Aug / 10. 1940. L. Gressitt [p, w]”; both were determined by Gressitt & Kimoto in 1962. 2♂♂, 2♀♀ (KMNH): “(Taiwan) / Alishan [阿里山] / Chiai Hsien [p, w] // May [p] 25 [h] .1971 [p] / K. Kanmiya [p, w]”; they were determined by Kimoto in 1973

#### Description.

**Figures 10–15.**
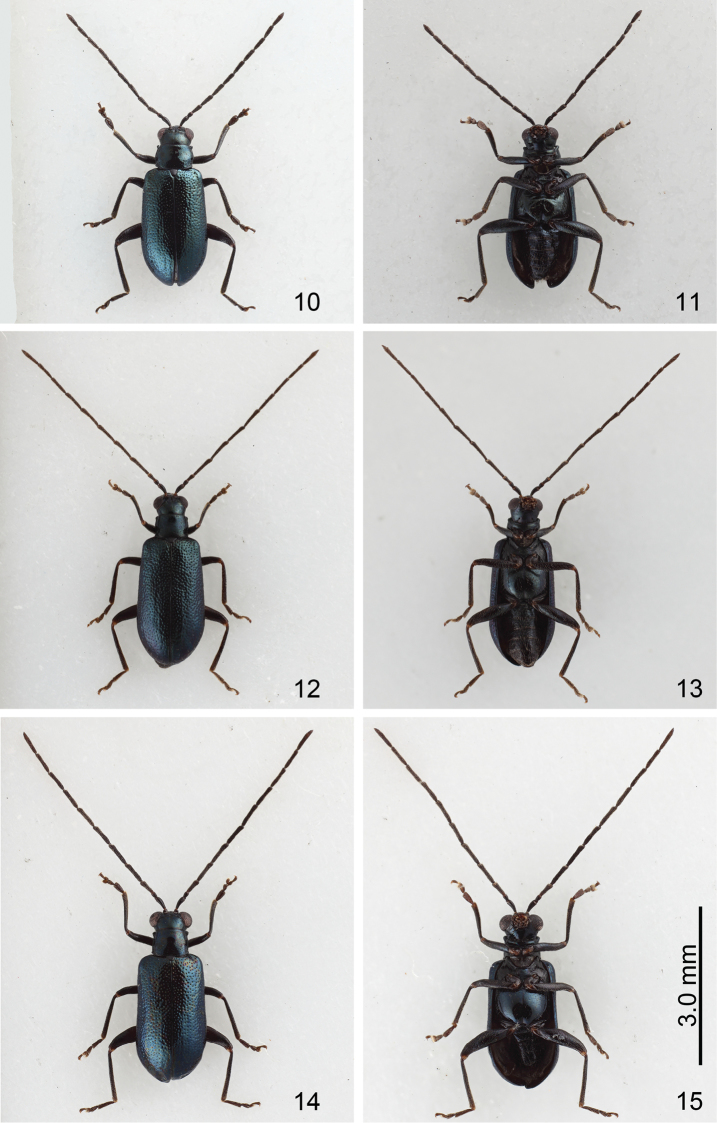
*Mandarella uenoi*, color variations, all at same scale. **10** Form A, dorsal view **11** Same, ventral view **12** Form B, dorsal view **13** Same, ventral view **14** Form C, dorsal view **15** Same, ventral view.

**Male.** Body size, relative lengths of antennomeres, and color patterns extremely variable, separated into six forms:

Form A (formerly identified as *Mandarella uenoi*): Length 3.1–3.5 mm. General color metallic blue except antenna, leg, and abdomen black (Figs 10–11). Elytron with longitudinal ridges. Antenna 0.8X as long as body, four apical antennomeres wide, ratio of length of antennomeres II to XI about 0.6 : 1.0 : 1.2 : 1.4 : 1.4 : 1.5 : 1.5 : 1.4 : 1.2 : 1.6; ratio of length to width from antennomeres II to XI about 1.8 : 3.0 : 3.6 : 4.0 : 3.7 : 3.4 : 2.7 : 3.0 : 2.9 : 3.9 (Fig. 20). Some individuals with longer antenna, as long as body, four apical antennomeres slender, ratio of length of antennomeres II to XI about 0.6 : 1.0 : 1.3 : 1.6 : 1.3 : 1.6 : 1.4 : 1.4 : 1.3 : 1.7; ratio of length to width from antennomere III to XI 1.8 : 3.0 : 4.0 : 4.7 : 4.1 : 4.6 : 4.2 : 4.2 : 3.9 : 4.1 (Fig. 21).

**Figures 16–19.**
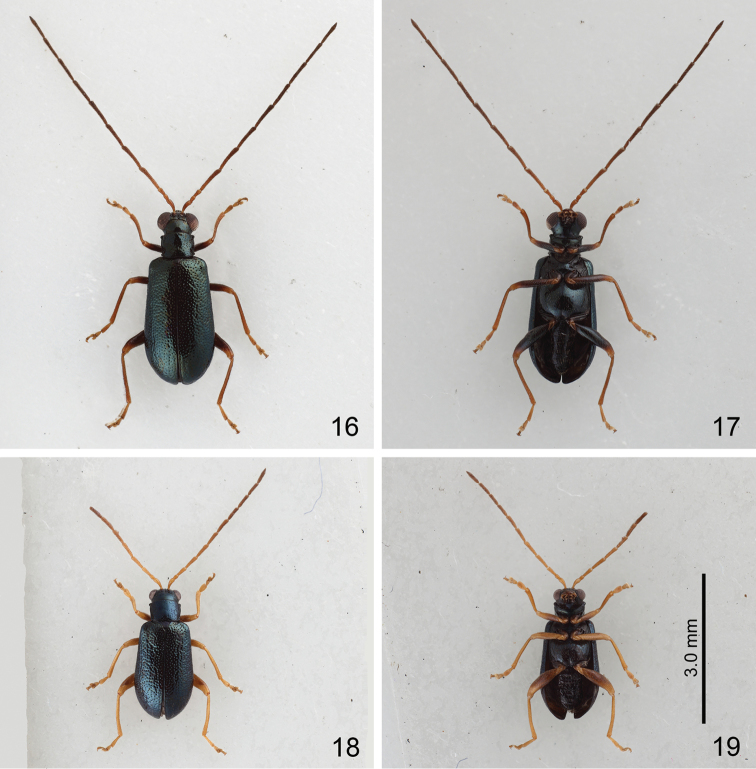
*Mandarella uenoi*, color variations, all at same scale. **16** Form D, dorsal view **17** Ditto, ventral view **18** Form E, dorsal view **19** Ditto, ventral view.

**Figures 20–32.**
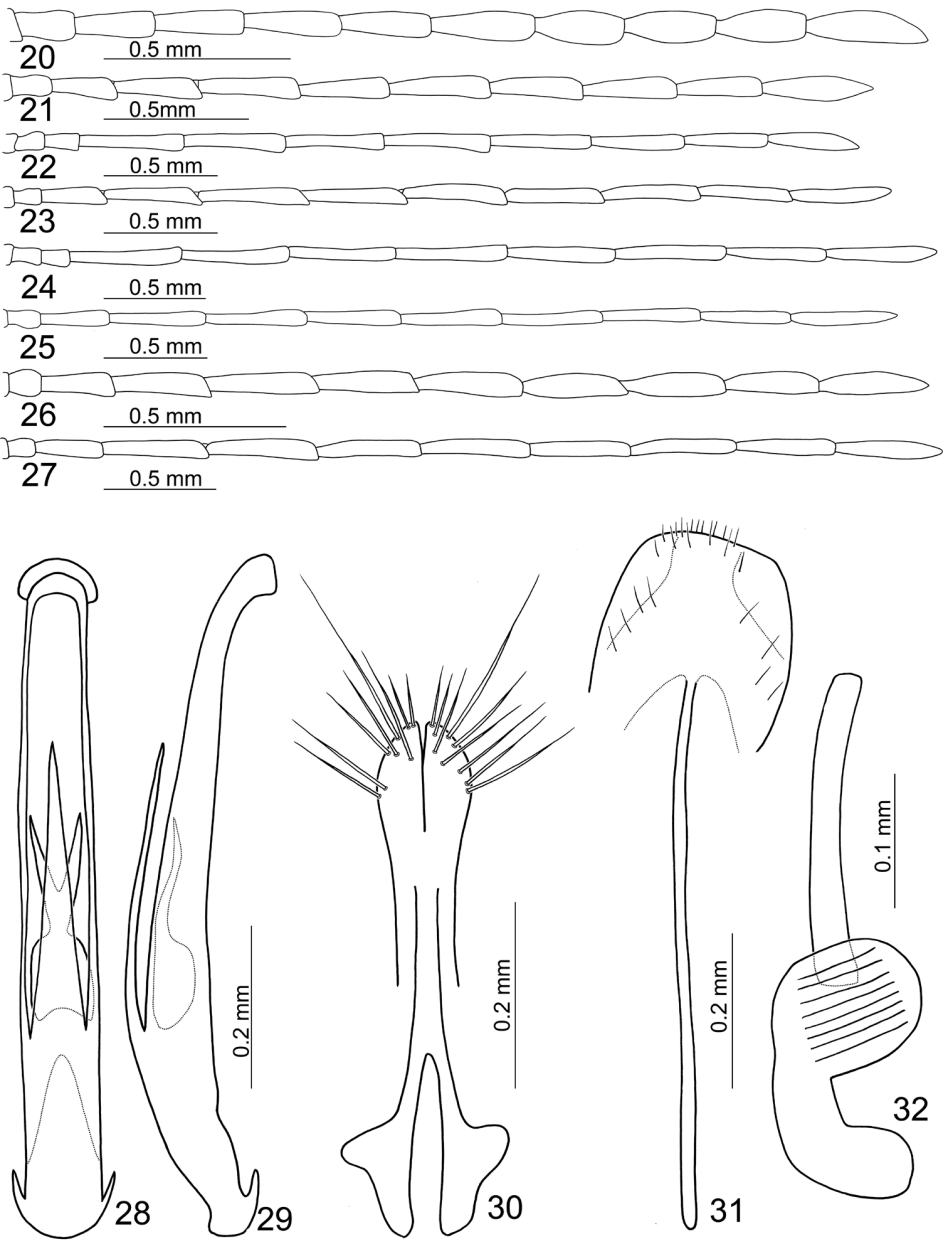
Diagnostic characters of *Mandarella uenoi*. **20** Male antenna, form A, typical **21** Male antenna, form A, elongate **22** Male antenna, form B **23** Male antenna, form C **24** Male antenna, form D, typical **25** Male antenna, form D, variation **26** Male antenna, form E **27** Male antenna, form F **28** Penis, dorsal view **29** Penis lateral view **30** Gonocoxae **31** Ventrite VIII **32** Spermatheca.

Form B (formerly identified as *Mandarella potanini*): Similar to form A, but body larger, length 3.7–4.1 mm; elytron without longitudinal ridges (Figs 12–13). Antennae longer, 1.2X longer than body, antennomere II subequal to III, antennomeres III-XI extremely slender, ratio of length of antennomeres II to XI about 0.7 : 1.0 : 2.6 : 2.7 : 2.5 : 2.5 : 2.5 : 2.3 : 2.2 : 2.3; ratio of length to width from antennomeres II to XI 1.6 : 2.2 : 6.2 : 6.5 : 6.2 : 6.1 : 6.1 : 6.6 : 6.2 : 6.5 (Fig. 22).

From C (formerly identified as *Mandarella nipponensis*): Length 3.8–4.1 mm. Similar to form B (Figs 14–15); antennae 1.2X longer than body, antennomere III much longer than II, III to XI extremely slender, ratio of length of antennomeres II to XI about 0.4 : 1.0 : 1.5 : 1.7 : 1.6 : 1.6 : 1.5 : 1.5 : 1.4 : 1.5; ratio of length to width from antennomere II to XI 1.7 : 4.1 : 6.1 : 6.9 : 7.0 : 7.0 : 6.8 : 6.7 : 6.4 : 6.8 (Fig. 23).

Form D (formerly identified as *Mandarella tibialis*): General color metallic blue but antennae and legs yellowish brown, coxa and femora metallic blue except apex (Figs 16–17). Length 3.4–3.7 mm. Antennae extremely long, about 1.5X longer than body, antennomere II subequal to III, III to X extremely slender; ratio of length of antennomeres II to XI 1.0 : 1.0 : 3.9 : 3.8 : 3.8 : 3.9 : 3.8 : 3.9 : 3.5 : 3.9; ratio of length to width from antennomeres II to XI 1.7 : 1.7 : 6.5 : 6.3 : 7.3 : 7.5 : 7.3 : 7.5 : 6.8 : 7.5 (Fig. 24). Some individuals with antennomere III much longer than II, III to X extremely slender; ratio of length of antennomeres II to XI 0.5 : 1.0 : 1.4 : 1.5 : 1.4 : 1.5 : 1.5 : 1.5 : 1.4 : 1.6; ratio of length to width from antennomeres II to XI 1.8 : 3.9 : 6.3 : 6.5 : 6.1 : 6.5 : 7.5 : 7.5 : 6.7 : 8.0 (Fig. 25).

Form E (formerly identified as *Mandarella kimotoi*): General color metallic blue but antennae and legs yellowish brown (Figs 18–19). Elytra with longitudinal ridges. Length 3.0–3.1 mm, antennae 0.9X as long as body, ratio of length of antennomeres II to XI about 0.5 : 1.0 : 1.3 : 1.6 : 1.5 : 1.5 : 1.5 : 1.4 : 1.3 : 1.6; ratio of length to width from antennomeres II to XI 1.2 : 2.9 : 4.1 : 4.8 : 4.5 : 4.7 : 4.5 : 4.2 : 4.1 : 4.8 (Fig. 26).

Form F (formerly identified as *Mandarella taiwana*): Similar to form E, but larger, length 3.4–3.7 mm. Antennae longer, about 1.1X longer than body; antennomere III much longer than III, III to X extremely slender; ratio of length of antennomeres II to XI 0.4 : 1.0 : 1.5 : 1.6 : 1.5 : 1.6 : 1.5 : 1.5 : 1.4 : 1.6; ratio of length to width from antennomeres II to XI 1.5 : 3.9 : 6.1 : 6.4 : 6.5 : 6.7 : 6.3 : 7.1 : 6.7 : 6.7 (Fig. 27).

Pronotum 1.4–1.6 times as broad long, quadrate, disc with scattered fine punctures, sometimes with feeble lateral depressions. Elytra 1.7–1.8 times as long as broad, parallel-sided, disc with dense, irregular, coarse punctures. First tarsomeres of front and middle legs extremely variable, extremely swollen, either elongate swollen or apically swollen. Posterior margin of last abdominal ventrite rounded, with two small incisions. Penis (Figs 28–29) extremely slender, about 9.1 times as long as broad; parallel-sided; tectum well sclerotized and apically tapering; almost straight in lateral view, curved near apex, apex truncate; endophallus with one longitudinal sclerite, apex bifurcate and forming two acute process, medially narrow.

**Females.** Length 3.5–3.7 mm, width 1.5–1.7 mm. Similar to male; head weakly constricted behind eyes. First tarsomeres of front and middle legs normal and not swollen. Gonocoxae (Fig. 30) slender, each gonocoxa apically widened, apex with eight setae; gonocoxae connected at middle, base abruptly and extremely widened. Ventrite VIII (Fig. 31) weakly sclerotized; apical margin with several short setae, disc with one pair of oblique dark stripes connected at apex, with several setae along outer margins of dark stripes; spiculum extremely long. Spermathecal receptaculum (Fig. 32) extremely swollen; pump strongly curved; sclerotized spermathecal duct long, weakly projecting into receptaculum.

#### Diagnosis.

**Figures 33–38.**
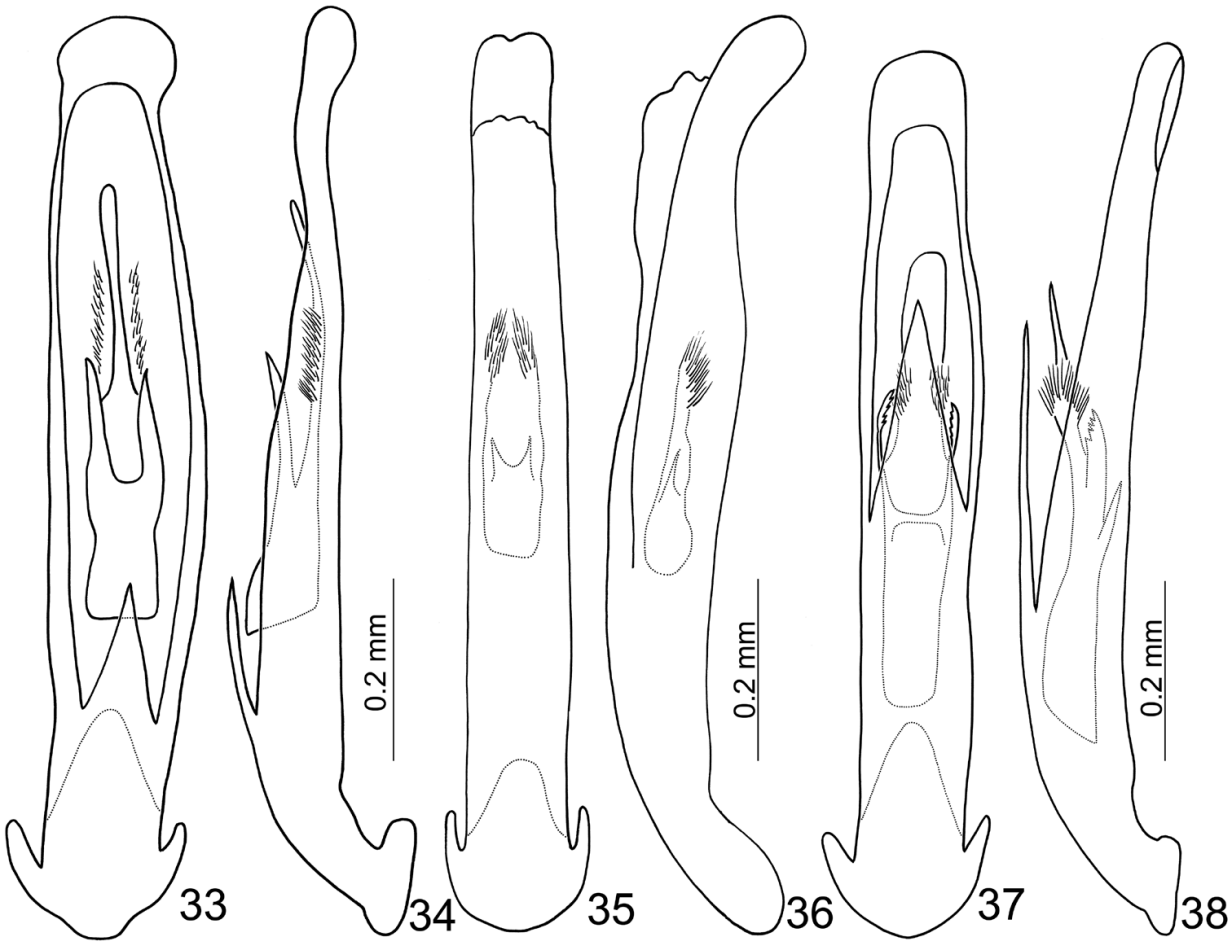
Penis of *Mandarella* species. **33** *Mandarella nipponensis*, dorsal view **34** Same, lateral view **35** *Mandarella tibialis*, dorsal view **36** Same, lateral view **37** *Mandarella potanini*, dorsal view **38** Same, lateral view.

*Mandarella nipponensis*, *Mandarella tibialis*, and *Mandarella potanini* were misidentified as *Mandarella uenoi*. *Mandarella nipponensis* possesses a wide and asymmetric penis (Figs 33–34). The penis of *Mandarella tibialis* is wider in lateral view with a pair of stout acute processes at the base of the endophallic sclerites (Figs 35–36). The penis of *Mandarella potanini* is wider and straight apically, and possesses a pair of serrate sclerites at the middle of the endophallic sclerites (Figs 37–38).

#### Host plants.

Adults are abundant at high altitudes during spring and summer. The first author collected more two hundred specimens in three hours in Yuanfeng, Nantou county on June 12, 2015. They were resting on leaves of various plants and produced very small feeding scars.

#### Distribution.

Endemic to Taiwan.

#### Other material examined.

A total of 1008 specimens was examined (Supplementary file 2: *Mandarella uenoi*, specimens examined).

### Mandarella tsoui sp. n.

Taxon classificationAnimaliaColeopteraChrysomelidae

http://zoobank.org/546B8B49-6115-4E64-A683-6B70DAFD8075

Stenoluperus pallipes : Kimoto, 1969: 39 (Taiwan); Kimoto, 1989: 253 (additional records in Taiwan). **Misidentification**

#### Type material of *Sternoluperus pallipes*.

Holotype ♂ (CAS): “Suisapa, 1000 M. / Lichuan Distr. / W. Hupeh, China / VII- [p] 26 [h] -48 [p, w] // Gressitt & / Djou Collrs. [p, w] // HOLOTYPE [p] / Stenoluperus / pallipes ♂ [h] // Gressitt & Kimoto [p, r] // Stenoluperus / pallipes / G & K [h] / J. L. Gressitt det. [p, w] // NO34 [p, w]”.

#### Type material

**(n = 354)**. Holotype ♂ (TARI): Nantou, Tsuifeng (翠峰), 2374 m, 21.IV.2015, leg. C.-F. Lee (TARI). Paratypes: **Chiayi**: 2♂♂, 1♀, Alishan (阿里山), 2216 m, 5-9.VIII.1981, leg. L. Y. Chou & S. C. Lin (TARI); 1♂, Fenchihu (奮起湖), 1400 m, 18.V.2014, leg. W.-C. Liao (TARI); 2♂♂, 5♀♀, Shihshan channel (石山引水道), 2300 m, 23.XI.2013, leg. W.-C. Liao (TARI); **Hsinchu**: 2♂♂, Kuanwu (觀霧), 2000 m, 30.IV.2010, leg. M.-H. Tsou (TARI); 1♂, Mamei (馬美), 1560 m, 18.V.2008, leg. S.-F. Yu (TARI); **Hualien**: 1♂, Kuanyuan (關原), 2374 m, 2.VII.2008, leg. M.-H. Tsou (TARI); 1♀, Piliu (碧綠), 2150 m, 13.VI.2014, leg. C.-F. Lee (TARI); 1♂, 1♀, Tayuling (大禹嶺), 2560 m, 9-16.VI.1980, leg. K. S. Lin & B. H. Chen (TARI); 1♂, same locality, 12-15.IX.1980, leg. K. S. Lin & C. H. Wang (TARI); 4♂♂, 1♀, same locality, 6-9.IX.1983, leg. L. Y. Chou & K. C. Chou (TARI); 1♀, Tzuen (慈恩), 2000 m, 12.VII.2014, leg. M.-H. Tsou (TARI); **Ilan**: 6♂♂, 2♀♀, Ssuyuanyakou (思源啞口), 1948 m, 28.IV.2009, leg. M.-H. Tsou (TARI); **Kaohsiung**: 3♀♀, Chungchihkuan (中之關), 1930 m, 16.IV.2012, leg. L.-P. Hsu; 1♀, same locality, 13.X.2012, leg. L.-P. Hsu (TARI); 1♀, Tengchih (藤枝), 1550 m, 2-5.VI.2008, leg. C.-F. Lee (TARI); 1♂, same locality, 26.V.2009, leg. C.-F. Lee (TARI); 3♂♂, 1♀, same locality, 13.IV.2013, leg. W.-C. Liao (TARI); 1♂, same locality, 8.VI.2013, leg. W.-C. Liao (TARI); 1♂, Tona (多納), 500 m, 3.II.2013, leg. W.-C. Liao (TARI); **Nantou**: 1♂, Chingching (清境), 1750 m, 27.VII.2013, leg. W.-C. Liao (TARI); 1♀, Meifeng (梅峰), 2100 m, 20-22.VI.1979, leg. K. S. Lin & B. H. Chen (TARI); 1♂, same locality, 26.VIII.1980, leg. K. S. Lin & C. H. Wang (TARI); 2♂♂, same locality, 28-29.VIII.1981, leg. L. Y. Chou & S. C. Lin (TARI); 1♂, 1♀, same locality, 8-11.V.1984, leg. K. C. Chou & C. C. Pan (TARI); 3♂♂, same locality, 17.VI.2010, leg. C.-F. Lee (TARI); 1♂, 2♀♀, same locality, 3.VII.2008, leg. M.-H. Tsou (TARI); 1♂, same locality, 20.IV.2011, leg. C.-F. Lee (TARI); 3♂♂, Nenkao trail (能高古道), 2600 m, 12.VII.2014, leg. J.-C. Chen (TARI); 2♂♂, 2♀♀, Nenkaoshan (能高山), 2860 m, 18.X.2011, leg. J.-C. Chen (TARI); 1♂, 3♀♀, Sungkang (松岡), 2000 m, 15-17.VIII.1984, leg. K. C. Chou (TARI); 1♂, 1♀, same locality, 13-15.IX.1984, leg. K. S. Lin & S. C. Lin (TARI); 1♂, same locality, 2.VII.2008, leg. M.-H. Tsou (TARI); 6♂♂, 9♀♀, Tatachia (塔塔加), 2610 m, 5.X.2008, leg. M.-H. Tsou (TARI); 1♂, 1♀, same locality, 9.VI.2009, leg. C.-F. Lee (TARI); 2♂♂, 1♀, 20.VII.2009, leg. S.-F. Yu (TARI); 1♂, same locality, 21.IX.2009, leg. M.-H. Tsou (TARI); 1♂, same locality, 30.X.2009, leg. C.-F. Lee (TARI); 4♂♂, 1♀, same locality, 17.XI.2009, leg. C.-F. Lee (TARI); 2♂♂, same locality, 19.XI.2009, leg. H. Lee (TARI); 2♂♂, same locality, 29.XII.2009, leg. M.-H. Tsou (TARI); 1♂, same locality, 27.IV.2010, leg. C.-F. Lee (TARI); 2♂♂, same locality, 9.VII.2014, leg. C.-F. Lee (TARI); 1♂, same locality, 13.VII.2014, leg. W.-C. Liao (TARI); 1♀, same locality, 1.VII.2015, leg. J.-C. Chen (TARI); 3♂♂, 2♀♀, Tsuifeng (翠峰), 2374 m, 21.VI.1979, leg. K. S. Lin & B. H. Chen (TARI); 3♂♂, 1♀, same locality, 3.VI.1980, leg. L. Y. Chou & C. C. Chen (TARI); 2♂♂, 5♀♀, same locality, 8.V.1981, leg. K. S. Lin & S. C. Lin (TARI); 15♂♂, 21♀♀, same locality, 25-27.VI.1981, leg. K. S. Lin & W. S. Tang (TARI); 2♂♂, 3♀♀, same locality, 1-3.VIII.1981, leg. T. Lin & W. S. Tang (TARI); 5♂♂, 1♀, same locality, 27.VIII.1981, leg. L. Y. Chou & S. C. Lin (TARI); 1♂, 1♀, same locality, 8.XI.1981, leg. S. C. Lin & W. S. Tang (TARI); 1♀, same locality, 23.V.1982, leg. L. Y. Chou (TARI); 7♂♂, 1♀, same locality, 1-3.IX.1982, leg. L.-Y. Chou & K. C. Chou (TARI); 2♂♂, same locality, 20.IV.1983, K. C. Chou & S. P. Huang (TARI); 3♂♂, same locality, 9.V.1984, leg. K. C. Chou & C. C. Pan (TARI); 2♂♂, 1♀, same locality, 5.VIII.1984, leg. K. S. Lin (TARI); 4♂♂, 6♀♀, same locality, 15-16.VIII.1984, leg. K. C. Chou (TARI); 1♂, same locality, 9.IV.2014, leg. C.-F. Lee (TARI); 1♂, Tungpu (東埔), 1200 m, 25-29.IX.1980, leg. L. Y. Chou & T. Lin (TARI); 7♂♂, 9♀♀, 28.IV.-2.V.1981, leg. T. Lin & C. J. Lee (TARI); 1♀, same locality, 22-25.XI.1982, leg. K. C. Chou & S. P. Huang (TARI); 1♂, 2♀♀, same locality, 20-24.VI.1983, leg. K. C. Chou & C. Y. Wong (TARI); 6♂♂, 3♀♀, same locality, 16-20.IV.1984, leg. K. C. Chou & C. H. Yung (TARI); 1♂, same locality, 23-27.VII.1984, leg. K. C. Chou & C. H. Yang (TARI); 1♀, Wushe (霧社), 1148 m, 30.VIII.-2.IX.1982, leg. L. Y. Chou & K. C. Chou (TARI); 13♂♂, 15♀♀, 19-22.IV.1983, leg. K. C. Chou & S. P. Huang (TARI); 1♂, 3♀♀, same locality, 7.V.1984, leg. K. C. Chou & C. C. Pan (TARI); **Pingtung**: 1♂, Jinshuiying (浸水營), 1450 m, 22.IX.2011, leg. J.-C. Chen (TARI); 1♀, Machia (瑪家), 1070 m, 17.III.2013, leg. W.-C. Liao (TARI); 1♂, Peitawushan (北大武山), 1100 m, 13.V.2010, leg. J.-C. Chen (TARI); 3♂♂, same locality, 21.III.2011, leg. J.-C. Chen (TARI); 1♂, same locality, 22.IX.2012, leg. J.-C. Chen (TARI); 1♂, Tahanshan (大漢山), 1200 m, 16.II.2013, leg. Y.-T. Chung (TARI); 2♂♂, Wutai (霧台), 1000 m, 22.III.2011, leg. J.-C. Chen (TARI); **Taichung**: 5♂♂, 3♀♀, Kukuan (谷關), 730 m, 14-17.X.1980, leg. K. S. Lin & C. H. Wang (TARI); 4♂♂, 13♀♀, Lishan (梨山), 2000 m, 26.VI.1979, leg. K. S. Lin & L. Y. Chou (TARI); 1♂, 2♀♀, Tahsuehshan (大雪山), 2600 m, 22.IX.2007, leg. M.-H. Tsou (TARI); 1♂, 1♀, Wuling (武陵), 1900 m, 27-29.VI.1979, leg. K. S. Lin & L. Y. Chou (TARI); **Taitung**: 1♀, Hsiangyang (向陽), 2320 m, 31.V.2011, leg. J.-C. Chen (TARI); 1♂, 1♀, same locality, 12.VII.2012, leg. J.-C. Chen (TARI); 1♂, 2♀♀, same locality, 9.V.2013, leg. J.-C. Chen (TARI); 2♀♀, same locality, 28.VI.2013, leg. J.-C. Chen (TARI); 1♂, 2♀♀, same locality, 22.XII.2013, leg. W.-C. Liao (TARI); 1♂, same locality, 28.III.2014, leg. J.-C. Chen (TARI); 1♀, same locality, 18.VII.2014, leg. W.-C. Huang (TARI); 4♂♂, 2♀♀, Liyuan (栗園), 1793 m, 8.VII.2010, leg. J.-C. Chen (TARI); 2♂♂, 1♀, 19.VI.2013, leg. B.-X. Guo (TARI); 5♂♂, 4♀♀, same locality, 19.VI.2013, leg. Y.-T. Chung (TARI); 1♂, same locality, 19.IV.2014, leg. W.-C. Huang (TARI); 7♂♂, 6♀♀, Motien (摩天), 1546 m, 5.X.2010, leg. C.-F. Lee (TARI); 1♂, same locality, 23.V.2011, leg. C.-F. Lee (TARI); 2♂♂, same locality, 19.VI.2011, leg. C.-F. Lee (TARI). 1♂ (EUMJ), labeled: “(TAIWAN) / Sungkang~ / Meifeng (2044~2127) / Nantow Co. / 18.V.1969 / S. Hisamatsu [p, w] // 松崗~梅峰 [h, w] // Sternoluperus / pallipes / Gressitt & Kimoto [h] / Det. S. Kimoto, 19 [p] 90 [h, w]”.

#### Description.

**Figures 39–44.**
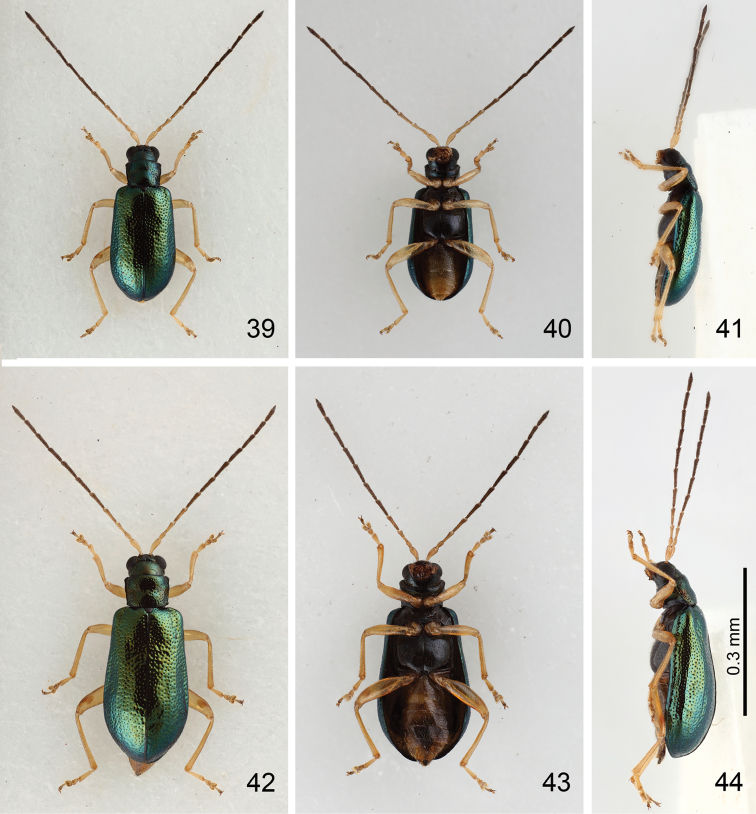
*Mandarella tsoui* sp. n., all at the same cale. **39** Male, dorsal view **40** Same, ventral view **41** Same, lateral view **42** Female dorsal view **43** Same, ventral view **44** Same, lateral view.

**Figures 45–51.**
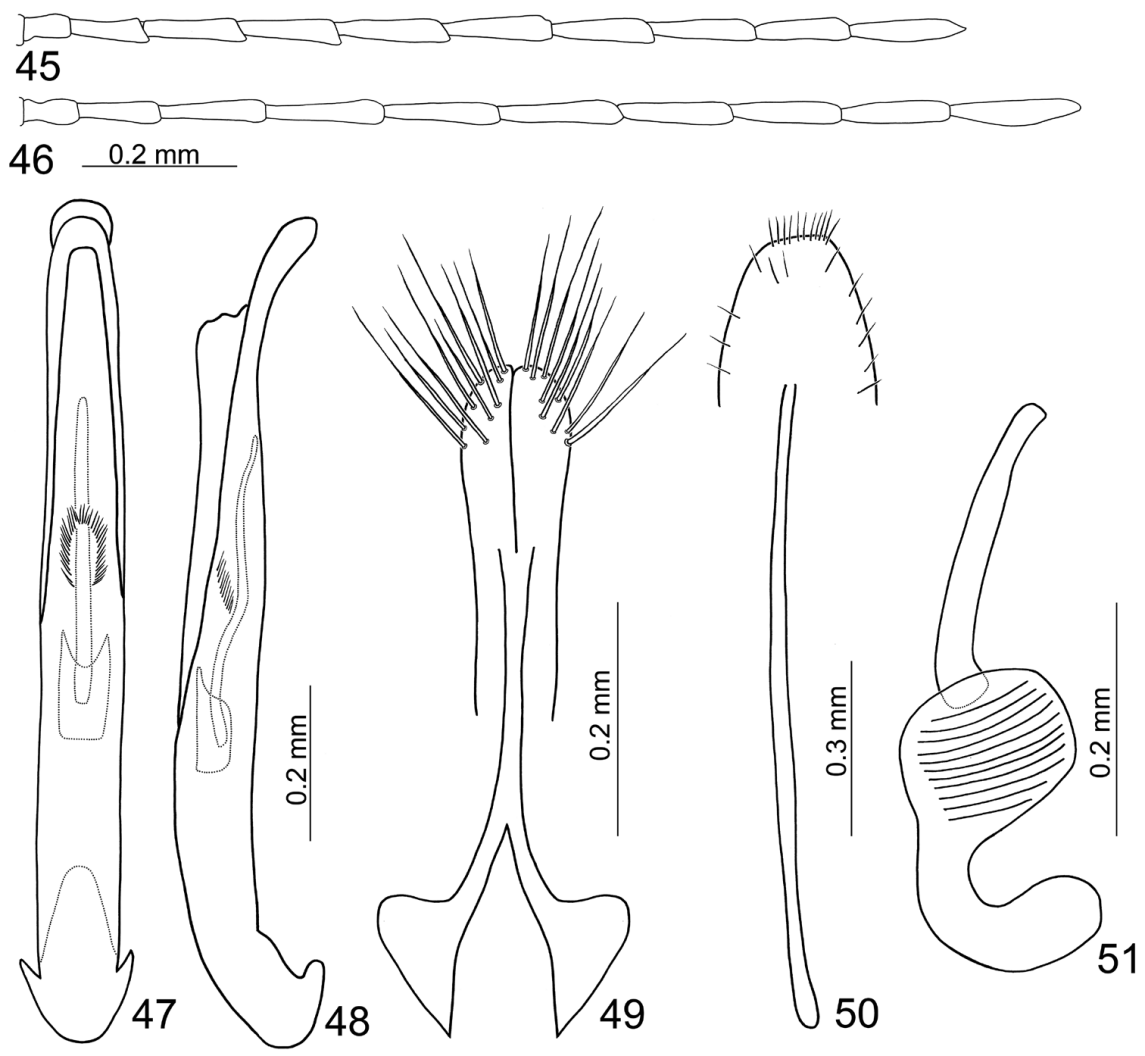
Diagnostic characters of *Mandarella tsoui* sp. n. **45** Male antenna, **46** Female antenna **47** Penis, dorsal view **48** Penis lateral view 49 Gonocoxae **50** Ventrite VIII **51** Spermatheca.

**Male.** Length 3.3–4.1 mm, width 1.3–1.7 mm. General color (Figs 39–41) bluish metallic; mouth parts, legs and abdomen yellowish; antennae dark brown but three or four basal antennomeres paler. Head weakly constricted behind eyes; antennae (Fig. 45) filiform and extremely slender, 1.2 times as body; ratio of length of antennomeres II to XI 0.6 : 1.0 : 1.3 : 1.3 : 1.5 : 1.4 : 1.3 : 1.4 : 1.3 : 1.6; ratio of length to width from antennomeres II to XI 2.0 : 3.2 : 4.3 : 4.2 : 4.7 : 4.6 : 4.3 : 4.4 : 4.0 : 5.3. Pronotum 1.3 times as broad long, quadrate, disc with scattered, coarse punctures, and with lateral depressions. Elytra 1.8 times as long as broad, parallel-sided, disc with dense, irregular, coarse punctures, and with depression at sides, and longitudinal ridge present along depression. First tarsomeres of front and middle legs swollen. Posterior margin of last abdominal ventrite rounded, with two small incisions, basal margin irregularly serrate. Penis (Figs 47–48) extremely slender, about 10.3 times as long as broad; parallel-sided; tectum membranous; almost straight in lateral view, weakly curved near apex, apex narrowly rounded; endophallus with one extremely elongate sclerite, sinuate in lateral view; base dorsally covered by one transverse sclerite.

**Female.** Length 4.3–4.7 mm, width 1.8–1.9 mm. Similar to male (Figs 42–44); ratio of length of antennomeres II to XI 0.6 : 1.0 : 1.3 : 1.5 : 1.4 : 1.5 : 1.4 : 1.4 : 1.3 : 1.6; ratio of length to width from antennomeres II to XI 2.1 : 3.6 : 4.8 : 5.4 : 5.2 : 5.4 : 5.1 : 4.9 : 4.9 : 5.0 (Fig. 46). First tarsomeres of front and middle legs normal and not swollen. Gonocoxae (Fig. 49) slender, each gonocoxa apically widened, apex with nine setae; gonocoxae connected at middle, base abruptly and extremely widened. Ventrite VIII (Fig. 50) weakly sclerotized; apical margin with several short setae, several long setae along lateral margin; spiculum extremely long. Spermathecal receptaculum (Fig. 51) extremely swollen; pump strongly curved; sclerotized spermathecal duct long, shallowly projecting into receptaculum.

#### Diagnosis.

This new species is similar to *Mandarella pallipes* but the latter lacks lateral depressions and ridges on the elytra.

**Host plant.** Adults are closely associated with *Stachyurus himalaicus* Hook. f. & Thomson ex Benth. (Stachyuraceae), which is sympatric with *Dercetina itoi* Kimoto, 1969 and *Dercetina shirozui* Kimoto, 1969.

#### Etymology.

This new species is named after Mr. Mei-Hua Tsou, a member of the TCRT and the first to collect this new species.

#### Distribution.

Endemic to Taiwan.

### Mandarella flaviventris

Taxon classificationAnimaliaColeopteraChrysomelidae

(Chen, 1942)

Stenoluperus flaviventris Chen, 1942: 67 (China: Jiangxi); Gressitt and Kimoto 1963: 580 (China: Fujian); Chûjô 1965: 98 (Taiwan); Kimoto 1969: 40 (additional records in Taiwan); Kimoto 1987: 189 (additional records in Taiwan); Kimoto 1989: 253 (additional records in Taiwan); Kimoto 1991c: 12 (additional records in Taiwan).
Stenoluperus esakii Kimoto, 1969: 40. **New synonym**
Stenoluperus matsumurai Takizawa, 1978: 128. **New synonym**
Mandarella matsumurai : Medvedev 2012: 427.
Stenoluperus itoi Kimoto, 1991b: 116 (nec Stenoluperus itoi Chûjô, 1966).
Mandarella taiwanensis Medvedev, 2012: 427 (replacement name for Stenoluperus itoi Kimoto, 1991). **New synonym**

#### Type material.

*Stenoluperus flaviventris*. The holotype was reportedly deposited at the Institute of Zoology, Chinese Academy of Sciences, China but could not be found (Yong-Ying Ruan, pers. comm. 8 October 2015).

*Sternoluperus esakii*. Holotype ♀ (KUEC): “[Formosa] / Hassenzan [八仙山] (Taichû-shû) / 13. Vii. 1932 / Teiso Esaki [p, w] // *Stenoluperus* / *esakii* / Kimoto, sp. n. [h, w] // HOLOTYPE [p, r]”.

*Stenoluperus itoi*. Paratype: 1♂ (KMNH): “Mt. YUSHAN [玉山] / TAIWAN / 19. V. 1981 / N. ITO [p, y] // *Stenoluperus* / *itoi* / Kimoto, sp. n. [h] / Det. S. Kimoto, 19 [p] 91 [h, w] // PARATOPOTYPE [p, b]”.

*Stenoluperus matsumurai*. Holotype ♂ (EIHU), holotype glued on the top of a triangular card; one front tibia and tarsi, antenna, and aedeagus also on the card: “Type [h, red letters, underside of triangular card] // Formosa / Matsumura [p, w] // (Japanese characters) / 21/IV1907 [h, underside of previous label] // Holo [h] –type [p] / *Stenoluperus* / *matsumurai* / Takizawa [h, r] // **Holotype** / Appended label by / ÔHARA, INARI, KANBE / SUZUKI and HIRONAGA / 2007 [p, w, with red band along right side] / 0000003054 / Sys. Ent / Hokkaido Univ. / Japan [SEHU] [p, w]. Paratypes: 1♂, 1♀, glued on tops of triangular cards, mounted with the same pin as holotype, the male has the blackish abdomen.

#### Voucher specimens.

1♀ (CAS): “FUKIEN, S. China / Shaowu: Tachulan / 1000 m. T. Maa [p, w] // Apr.30.1942 [h, w]”; 1♂, 1♀ (CAS): “FUKIEN, S. China / Shaowu: Tachulan / 1000 m. T. Maa [p, w] // Apr.17,1943 [h, w]”; 1♂ (BPBM): “FUKIEN, S. China / Shaowu: Tachulan [p] / IV.20. [h] 194 [p] 3 [h] T. Maa [p, w] // N52 [h, w] // ILL [p, w]”; 4♂♂ (BPBM), 2♀♀ (CAS): “FUKIEN, S. China / Shaowu: Tachulan / 1000 m. T. Maa [p, w] // Apr.27,1943 [h, w]”; they were determined by Gressitt & Kimoto in 1962. 1♂ (KMNH): “ (Taiwan) / Alishan, [阿里山] 2300m / Chiayi Hsien [p, w] // 9. [h] iv.1965 [p] / Y. Hirashima [p, w] // Japan-U. S. / Co-op. Sci. / Programme [p, y]”; 1♀ (KMNH): “ (Taiwan) / Meifeng [梅峰] / Nantou Hsien [h, w] // 18.V.1965 / B. S. Chang [h, w]”; 1♂ (CAS): “FORMOSA: / Arisan. [阿里山] / VIII-18-1947 / J. L. Gressitt [p, w] // L. Gressitt / Collection [p, w]”; they were determined by Kimoto in 1968. 1♂ (KMNH): “ (Taiwan) / Alishan [阿里山] / Hsien [p, w] // May [p] 25 [h] .1971 [p] / K. Kanmiya [p, w]”; it was determined by Kimoto in 1973. 1♂ (KMNH): “ALISHAN [阿里山] / TAIWAN / 3. V. 1983 / T. ITO [p, y]”, it was determined by Kimoto in 1987. 1♀ (EUMJ): “(TAIWAN) / Sungkang~ / Meifeng (2044~2127) / Nantow Co. / 18.V.1969 / S. Hisamatsu [p, w] // 松崗~梅峰 [h, w]”; it was determined by Kimoto in 1991.

#### Description.

**Figures 52–57.**
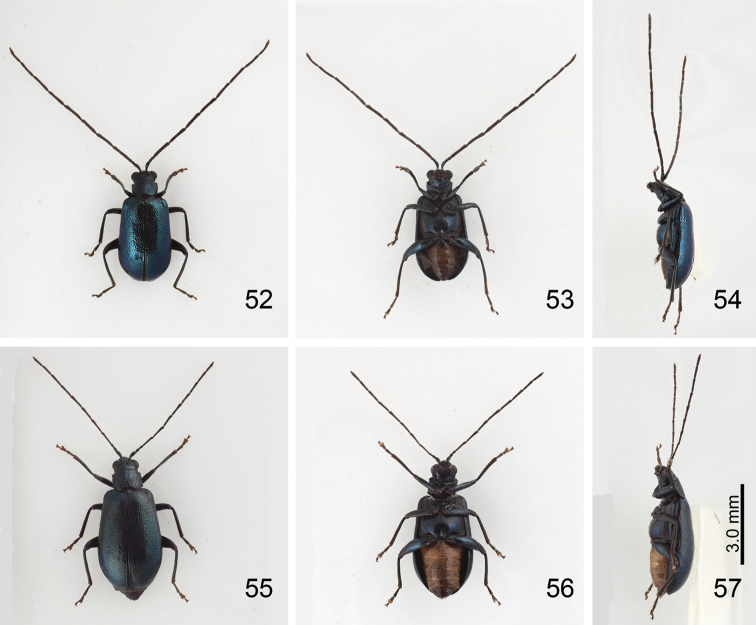
*Mandarella flaviventris*, form G, all at same cale. **52** Male, dorsal view **53** Same, ventral view **54** Same, lateral view **55** Female dorsal view **56** Same, ventral view **57** Same, lateral view.

Color patterns and relative lengths of antennae separated into four forms:

Form G (formerly identified as *Mandarella flaviventris*): General color (Figs 52–57) bluish metallic; antennae black and abdomen yellow. In male, antennae (Fig. 62) filiform and extremely slender, 1.4 times as body; ratio of length of antennomeres II to XI 1.2 : 1.0 : 4.5 : 5.0 : 5.0 : 5.4 : 5.2 : 5.2 : 4.5 : 5.0; ratio of length to width from antennomeres II to XI 1.5 : 1.1 : 5.0 : 5.6 : 5.6 : 6.0 : 5.8 : 5.8 : 5.7 : 6.4. In female, antennae shorter, as long as body (Fig. 63), antennomeres III relatively longer, ratio of length of antennomeres II to XI 0.9 : 1.0 : 2.6 : 2.8 : 2.6 : 2.7 : 2.4 : 2.4 : 2.1 : 2.4; ratio of length to width from antennomeres II to XI about 1.8 : 2.0 : 5.3 : 5.8 : 5.4 : 5.6 : 5.3 : 5.5 : 5.0 : 5.2.

**Figures 58–61.**
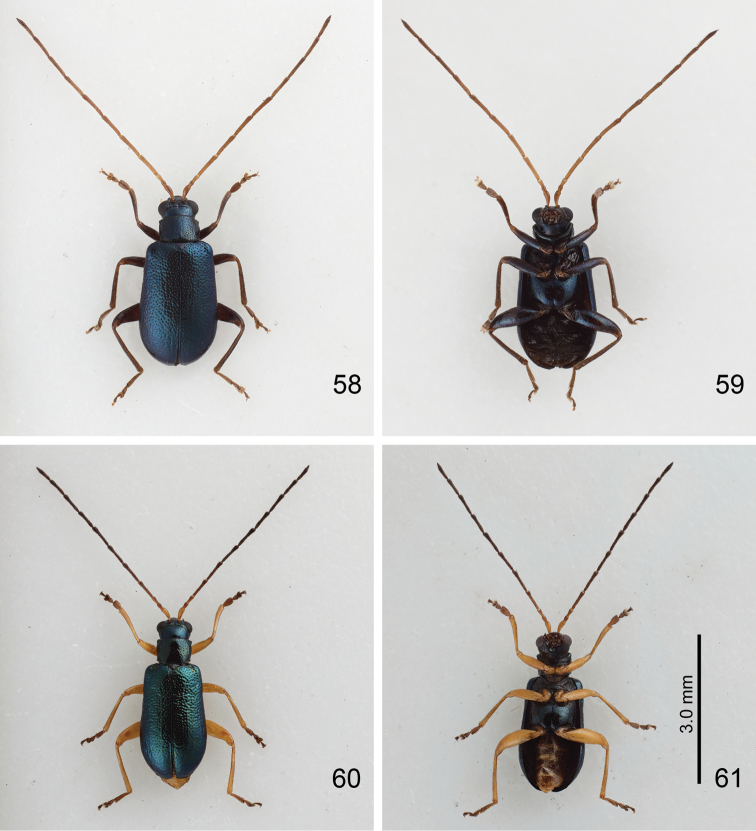
*Mandarella flaviventris*, color variation, all at same scale. **58**. Form H, male, dorsal view **59** Same, ventral view **60** Form I, male, dorsal view **61** Same, ventral view.

**Figures 62–71.**
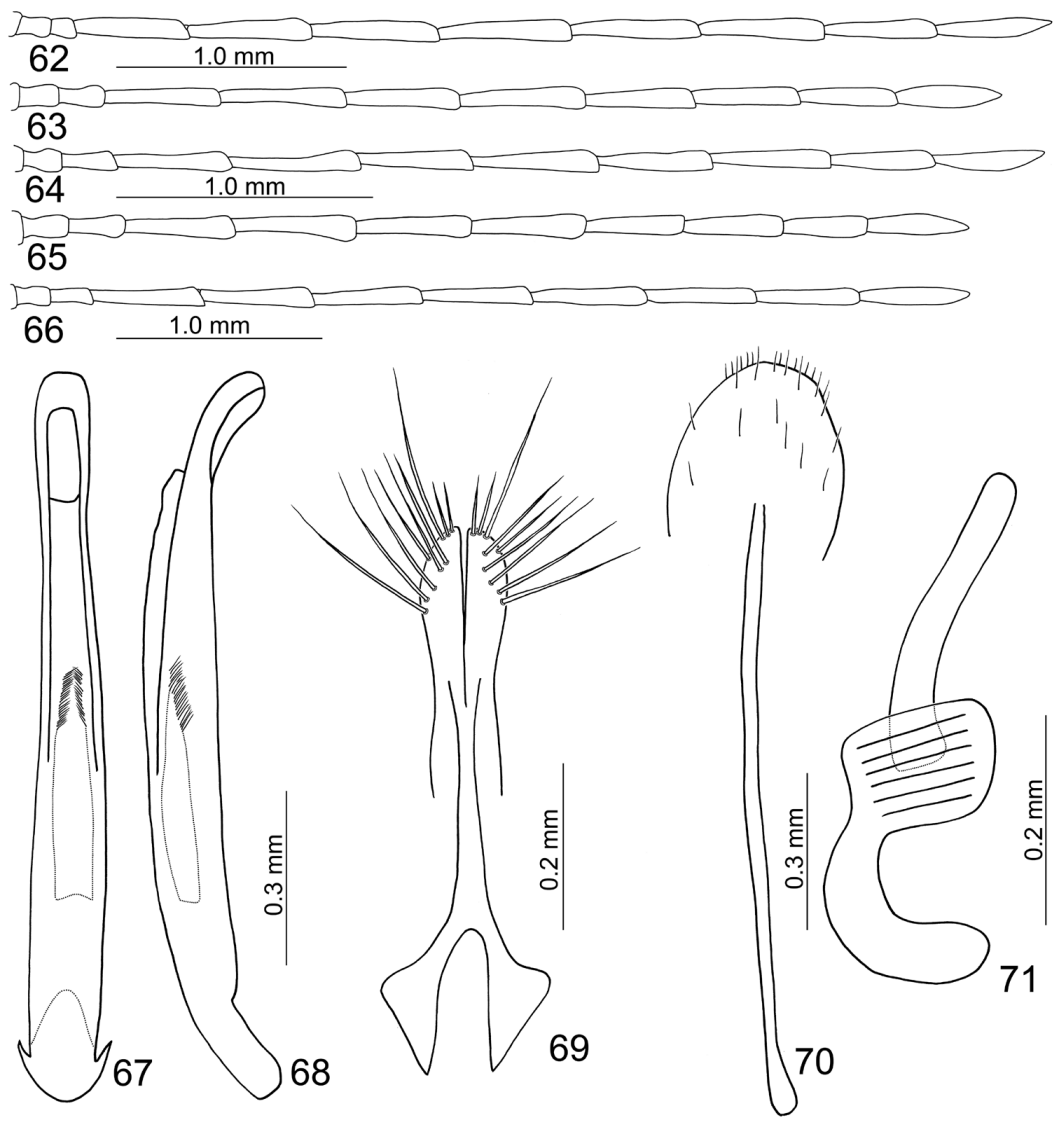
Diagnostic characters of *Mandarella flaviventris*. **62** Male antenna, form G **63** Female antenna, form G **64** Male antenna, form I **65** Female antenna form I **66** Male antenna, form J **67** Penis, dorsal view **68** Same, lateral view **69** Gonocoxae **70** Ventrite VIII **71** Spermatheca.

Form H (formerly identified as *Mandarella esakii*): Similar to form G, but antennae and legs dark brown (Figs 58–59).

Form I (formerly identified as *Mandarella matsumurai*): General color metallic blue but antennae, legs, and abdomen yellowish brown; seven apical antennomeres darkened antennomeres (Figs 60–61). In male, antennae (Fig. 64) 1.3× longer than body, antennomere II a little longer than III, III to X extremely slender, ratio of length of antennomeres II to XI 0.7: 1.0 : 2.1 : 2.4 : 2.2 : 2.3 : 2.1 : 2.2 : 1.9 : 2.1; ratio of length to width from antennomeres II to XI 1.7 : 2.6 : 5.6 : 6.2 : 5.7 : 6.1 : 5.6 : 5.8 : 5.0 : 5.4. In female, antennae shorter (Fig. 65), 0.9 times as long as body, antennomeres III relatively longer, ratio of length of antennomeres II to XI 0.8 : 1.0 : 2.0 : 2.2 : 2.1 : 2.1 : 1.8 : 1.8 : 1.5 : 1.9; ratio of length to width from antennomeres II to XI 2.0 : 2.4 : 4.7 : 5.3 : 5.1 : 4.9 : 4.5 : 4.5 : 3.9 : 4.7.

Form J (formerly identified as *Mandarella taiwanensis*): Color pattern similar to form I, but abdomen blackish brown. In male, antennae 1.3X longer than body (Fig. 66), antennomere II a little longer than III, III to X extremely slender, ratio of length of antennomeres II to XI about 0.8 : 1.0 : 2.8 : 2.8 : 2.7 : 2.7 : 2.8 : 2.7 : 2.5 : 2.7; ratio of length to width from antennomeres II to XI 1.7 : 2.3 : 6.4 : 6.4 : 6.1 : 6.1 : 6.4 : 6.1 : 5.7 : 6.1.

**Male.** Length 3.6–4.6 mm, width 1.6–2.1 mm. Head strongly constricted behind eyes. Pronotum 1.4 times as broad long, quadrate, disc with dense and coarse punctures as on elytra, lacking lateral depressions. Elytra 1.7 times as long as broad, parallel-sided, disc with dense, irregular, coarse punctures. First tarsomeres of front and middle legs swollen. Posterior margin of last abdominal ventrite truncate, with two small incisions. Penis (Figs 67–68) extremely slender, about 10.1 times as long as broad; parallel-sided; tectum membranous; almost straight in lateral view, weakly curved near apex, apex narrowly rounded, ventral disc depressed near apex; endophallus with one elongate sclerite, weakly sclerotized, with dense setae above middle, and small teeth at middle; straight in lateral view.

**Female.** Length 3.9–5.4 mm, width 1.9–2.6 mm. Similar to male; but head weakly constricted behind eyes. First tarsomeres of front and middle legs normal and not swollen. Gonocoxae (Fig. 69) slender, each gonocoxa apically widened, apex with nine setae; gonocoxae connected at middle, base abruptly and extremely widened. Ventrite VIII (Fig. 70) weakly sclerotized; apical margin with several short setae, disc with several long setae scattered; spiculum extremely long. Spermathecal receptaculum (Fig. 71) extremely swollen; pump strongly curved; sclerotized spermathecal duct long, deeply projecting into receptaculum.

#### Diagnosis.

Although *Mandarella flaviventris* is highly variable in color patterns, it is characterized by the small third antennomere (3^rd^ antennomeres ≤1.3 times as long as 2^nd^ antennomere). Some black individuals of *Mandarella uenoi* also have small 3^rd^ antennomeres, similar to *Mandarella flaviventris* but their abdomens are black (yellow abdomens in *Mandarella flaviventris*).

#### Host plants.

Like *Mandarella uenoi*, adults rested on leaves of various plants and left small feeding scars.

#### Distribution.

China (Fujian, Jiangxi), Taiwan.

#### Other material examined.

Totally 717 specimens were studied (Suppl. material 3: *Mandarella flaviventris*, specimens examined).

### Key to the Taiwanese species of *Mandarella* Duvuvier

**Table:** 

1	Lateral depression and ridge present on each elytron; antennae, legs, and abdomen yellow, and antennomere III much longer than antennomere II (1.6 times)	***Mandarella tsoui* sp. n.**
–	Lateral depression and ridge absent from each elytron; individuals with yellow antennae, legs, and abdomen, antennomere III slightly longer than antennomere II (1.3 times) (form I of *Stenoluperus flavivientris*)	**2**
2	Individuals with black or blackish legs, abdomen black, in males antennomere III from slightly longer to much longer than antennomeres II (≥1.3 times); individuals with yellow legs, antennomere III much longer than antennomere II (≥2.0 times); tectum of penis apically tapering, apex of endophallic sclerite bifurcate and acute	***Mandarella uenoi* (Kimoto, 1969)**
–	Individuals with black or blackish legs, abdomen yellow, in males antennomere III shorter than antennomere II (0.8 times); individuals with yellow, antennomere III slightly longer than antennomere II (1.3 times); tectum of penis membranous and invisible, apex of endophallic sclerite with dense marginal setae	***Mandarella flaviventris* (Chen, 1942)**

## Discussion

A total of 11 species within *Mandarella* has been reported from Taiwan. Molecular analyses based on the COI sequences and morphological studies, including male aedeagi, revealed that only three species exist in Taiwan (Fig. 9). Variations exist in color patterns and ratios of the lengths between the antennomeres II and III, and these two morphological characteristics can be employed as diagnostic characters, as shown in identification key mentioned above, to distinguish the three major lineages. But, non-overlapping genetic distances of COI, i.e. >16.2% (interspecies) and <14.4% (intraspecies), for the three flea beetle lineages also are useful for delineation.

Morphological characters (i.e., body sizes, color patterns, relative lengths between antennomeres) used previously to identify different morphospecies likely are a reflection of their altitudinal distributions induced by local adaptations. For example, in the Hohuanshan mountains, the black forms A/B was mainly collected at an elevation of 3422 m (Hohuanshan Mt. Peak), while at 2756 m (Yuanfeng), the form D with yellow legs and darkened basal femora, is dominant with only seven out of 239 specimens representing the form A. Phylogenetic inferences of these *Mandarella* flea beetles also revealed local adaptation. The variable morphological forms recognized in the *Mandarella uenoi* and *Mandarella flaviventris* lineages may represent a more complicated scenario and related to evolutionary processes. Additional specimens from different montane areas are required to elucidate their phylogeographic histories and address the local adaptations of body size, body color, and the length of antennomeres.

## Supplementary Material

XML Treatment for Mandarella uenoi

XML Treatment for Mandarella tsoui

XML Treatment for Mandarella flaviventris

## Supplementary materials

Supplementary material 1 Collection information, taxon ID, and accession numbers of COI gene for each flea beetle Data type: DNA alignment Explanation note: DNA Submission in GenBank. This dataset is made available under the Open Database License (http://opendatacommons.org/licenses/odbl/1.0/). The Open Database License (ODbL) is a license agreement intended to allow users to freely share, modify, and use this Dataset while maintaining this same freedom for others, provided that the original source and author(s) are credited.Chi-Feng Lee, Cheng-Lung Tsai, Alexander Konstantinov, Wen-Bin Yeh

Supplementary material 2
*Mandarella uenoi*, other material examined Data type: Occurence Explanation note: 1008 specimens were examined. The localities, dates, and depositories were recorded. This dataset is made available under the Open Database License (http://opendatacommons.org/licenses/odbl/1.0/). The Open Database License (ODbL) is a license agreement intended to allow users to freely share, modify, and use this Dataset while maintaining this same freedom for others, provided that the original source and author(s) are credited.Chi-Feng Lee, Cheng-Lung Tsai, Alexander Konstantinov, Wen-Bin Yeh

Supplementary material 3
*Mandarella flaviventrites*, other material examined Data type: Occurence Explanation note: 707 specimens were examined. The localities, dates, and depositories were recorded. This dataset is made available under the Open Database License (http://opendatacommons.org/licenses/odbl/1.0/). The Open Database License (ODbL) is a license agreement intended to allow users to freely share, modify, and use this Dataset while maintaining this same freedom for others, provided that the original source and author(s) are credited.Chi-Feng Lee, Cheng-Lung Tsai, Alexander Konstantinov, Wen-Bin Yeh
