# Supplementary material for: Revision of Mandarella Duvivier from Taiwan, with a new species, new synonymies and identities of highly variable species (Insecta, Chrysomelidae, Galerucinae, Alticini)
Source: Zookeys. 2016 Feb 23;(568):23–49. doi: 10.3897/zookeys.568.7125 (PMC4829668; doi:10.3897/zookeys.568.7125)
Supplement: Supplementary material 2 — Mandarella uenoi, other material examined [file zookeys-568-023-s002.pdf]

Other Material examined. Form A (n= 410). **Hsinchu**: 2♀♀, Kuanwu (觀霧), 2000 m, 30.IV.2010, leg. M.-H. Tsou (TARI); **Hualien**: 5♀♀, Piliu (碧綠), 2150 m, 31.V.2011, leg. M.-H. Tsou (TARI); 1♀, same locality, 13.VI.2014, leg. C.-F. Lee (TARI); 1♂, 1♀, Tayuling (大禹嶺), 2560 m, 9-16.VI.1980, leg. K. S. Lin & B. H. Chen (TARI); **Ilan**: 5♀♀, Taipingshan (太平山), 1950 m, 3.VII.2010, leg. M.-H. Tsou (TARI); 2♀♀, same locality, 24.VI.2012, leg. M.-H. Tsou (TARI); **Nantou**: 1♀, Hohuanshan (合歡山), 3422 m, 18.V.2009, C.-F. Lee (TARI); 2♀♀, same locality, 26.VII.2014, log. J.-C. Chen (TARI); 1♂, 2♀♀, Hsiaofengkou (小風口), 3002 m, 9.VIII.2012, leg. C.-F. Lee (TARI); 5♀♀, Nenkao trail (能高古道), 2600 m, 12.VII.2014, leg. J.-C. Chen (TARI); 1♂, 2♀♀, Patungkuan (八通關), 2800 m, 13-15.VI.1989, leg. K. W. Huang (NMNS); 1♂, same locality, 20-22.VI.1990, leg. J. T. Yang (NMNS); 3♀♀, Tatachia (塔塔加), 2610 m, 20.VII.2009, leg. C.-F. Lee (TARI); 1♀, same locality, 27.IV.2010, leg. C.-F. Lee (TARI); 1♂, Tsuifeng (翠峰), 2374 m, 18.VI.2010, leg. C.-F. Lee (TARI); 1♂, same locality, 11.VI.2014, leg. C.-F. Lee (TARI); 12♂♂, 6♀♀, Wuling (武嶺), 3275 m, 25.VI.2012, leg. C.-F. Lee (TARI); 12♂♂, 4♀♀, Yuanfeng (鳶峰), 2756 m, 9-19.V.1998, leg. C. S. Lin & W. T. Yang (NMNS); 2♂♂, 5♀♀, 12.VI.2014, leg. C.-F. Lee (TARI); **Taichung**: 11♂♂, 2♀♀, Paikutashan (白姑大山), 3341 m, 21.VI.2014, leg. T.-H. Lee (TARI); 19♂♂, 15♀♀, Hsuehshan (雪山), 3886 m, 8-14.VI.2008, leg. W.-B. Yeh (TARI); 4♀♀, same locality, 7.VIII.2008, leg. W. T. Yang (NMNS); 17♂♂, 10♀♀, same locality, 26-29.V.2009, leg. W.-B. Yeh (TARI); 4♀♀, same locality, 5.VIII.2009, leg. W.-B. Yeh (TARI); 3♀♀, same locality, 5.IX.2009, leg. W.-B. Yeh (TARI); 31♂♂, 55♀♀, same locality, 17-18.VI.2010, leg. W.-B. Yeh (TARI); 3♀♀, same locality, 3-4.VIII.2010, leg. W.-B. Yeh (TARI); 6♂♂, 6♀♀, same locality, 9-10.VI.2011, leg. W.-B. Yeh (TARI); 1♂, 10♀♀, same locality, 2-3.VIII.2011, leg. W.-B. Yeh (TARI); 1♂, same locality, 7.X.2011, leg. W.-B. Yeh (TARI); 43♂♂, 75♀♀, same locality, 29.IV.-28.VI.2012, leg. L.-P. Hsu (TARI); 1♀, same locality, 2.V.2012, leg. T.-H. Lee (TARI); 2♀♀, Tahsuehshan (大雪山), 2600 m, 5.VI.2012, leg. J.-C. Chen (TARI); **Taitung**: 1♂, Hsiangyang (向陽), 2320 m, 9.V.2013, leg. J.-C. Chen (TARI); 1♂, 8♀♀, Hsiangyangshan (向陽山), 3603 m, 20.VI.2014, leg. J.-C. Chen (TARI); 1♀, Motien (摩天), 1546 m, 23.V.2011, leg. C.-F. Lee (TARI); 1♀, same locality, 19.VI.2011, leg. C.-F. Lee (TARI).

Form B (n= 222). **Hualien**: 1♀, Piliu (碧綠), 2150 m, 17.V.2009, leg. C.-F. Lee (TARI); 1♂, 3♀♀, Tayuling (大禹嶺), 2560 m, 9-16.VI.1980, leg. K. S. Lin & B. H. Chen (TARI); **Ilan**: 2♂♂, Taipingshan (太平山), 1950 m, 26-28.VII.1983, leg. L. Y. Chou (TARI); **Nantou**: 2♂♂, 2♀♀, Hohuanshan (合歡山), 3422 m, 2.VII.2008, leg. M.-H. Tsou (TARI); 1♂, 1♀, same locality, 2.VII.2008, leg. S.-F. Yu (TARI); 4♂♂, 4♀♀, same locality, 17-18.V.2009, leg. C.-F. Lee (TARI); 2♂♂, same locality,

17.V.2009, leg. M.-H. Tsou (TARI); 5♂♂, 16♀♀, same locality, 6.VII.2015, leg. C.-F. Lee (TARI); 1♀, Hsiaofengkou (小風口), 3002 m, 31.VII.-1.VIII.2007, leg. Lin, Chan, Wu & Hou (NMNS); 1♀, Meifeng (梅峰), 2100 m, 2-4.VI.1980, 28-29.VIII.1981, leg. L. Y. Chou & S. C. Lin (TARI); 1♀, Nenkao trail (能高古道), 2600 m, 12.VII.2014, leg. J.-C. Chen (TARI); 1♂, Tatachia (塔塔加), 2610 m, 9.VI.2009, leg. C.-F. Lee (TARI); 5♂♂, 5♀♀, Tsuifeng (翠峰), 2374 m, 3.VI.1980, leg. L. Y. Chou & C. C. Chen (TARI); 3♂♂, 10♀♀, same locality, 25-27.VI.1981, leg. K. S. Lin & W. S. Tang (TARI); 1♂, same locality, 23.V.1982, leg. L. Y. Chou (TARI); 1♂, same locality, IV.1984, leg. K. S. Lin & K. C. Chou (TARI); 1♂, same locality, 18.VI.2010, leg. C.-F. Lee (TARI); 1♀, same locality, 6.VII.2015, leg. C.-F. Lee (TARI); 1♂, 1♀, Wuling (武嶺), 3275 m, 25.VI.2012, leg. C.-F. Lee (TARI); 21♂♂, 16♀♀, Yuanfeng (鳶峰), 2756 m, 12.VI.2014, leg. C.-F. Lee (TARI); **Taichung**: 11♂♂, 8♀♀, Hsuehshan (雪山), 3886 m, 8.VI.2008, leg. W.-B. Yeh (TARI); 5♂♂, 2♀♀, same locality, 29.V.2009, leg. W.-B. Yeh (TARI); 2♀♀, same locality, 5.VIII.2009, leg. W.-B. Yeh (TARI); 32♂♂, 19♀♀, same locality, 17-18.VI.2010, leg. W.-B. Yeh (TARI); 2♂♂, same locality, 3.VIII.2010, leg. W.-B. Yeh (TARI); 18♂♂, 9♀♀, same locality, 9.VI.2011, leg. W.-B. Yeh (TARI); 1♂, 2♀♀, same locality, 2.VIII.2011, leg. W.-B. Yeh (TARI); 4♀♀, Kukuan (谷關), 730 m, 14-17.X.1980, leg. K. S. Lin & C. H. Wang (TARI); **Taitung**: 1♂, Hsiangyangshan (向陽山), 3603 m, 20.VI.2014, leg. J.-C. Chen (TARI); 2♀♀, Liyuan (栗園), 1793 m, 19.VI.2013, leg. C.-F. Lee (TARI).

Form C (n= 285). **Chiayi**: 1♀, Alishan (阿里山), 2216 m, 12.V.2011, leg. C.-F. Lee (TARI); 1♀, Tzuchung (自忠), 2280 m, 8.V.2015, leg. J.-C. Chen (TARI); **Hsinchu**: 2♀♀, Kuanwu (觀霧), 2000 m, 30.IV.2010, leg. M.-H. Tsou (TARI); 6♂♂, Lupi (魯壁), 1500 m, 4.IV.2009, leg. M.-H. Tsou (TARI); 2♂♂, same locality, 18.IV.2009, leg. M.-H. Tsou (TARI); 1♀, same locality, 12.IV.2015, leg. M.-H. Tsou (TARI); 1♂, 2♀♀, same locality, 19.IV.2015, leg. M.-H. Tsou (TARI); **Hualien**: 1♂, 1♀, Piliu (碧綠), 2150 m, 17.V.2009 leg. C.-F. Lee (TARI); **Ilan**: 1♀, Ssuyuanyakou (思源啞口), 1948 m, 28.IV.2009, leg. M.-H. Tsou (TARI); 1♂, Taipingshan (太平山), 1950 m, 30.IV.2009, leg. C.-F. Lee (TARI); **Kaohsiung**: 7♂♂, 2♀♀, Tianchi (天池), 2280 m, 31.III.2015, leg. C.-F. Lee (TARI); 1♂, 3♀♀, Kuanshanyakou (關山啞口), 2930 m, 30.VII.2015, leg. C.-F. Lee (TARI); **Nantou**: 1♀, Meifeng (梅峰), 2100 m, 2-4.VI.1980, leg. L. Y. Chou & C. C. Chen (TARI); 1♀, same locality, 8-11.V.1984, leg. K. C. Chou & C. C. Pan (TARI); 1♂, same locality, 13.VI.-18.VII.2001, leg. C. S. Lin & W. T. Yang (NMNS); 1♂, 1♀, same locality, 11.VI.-8.VII.2003, leg. C. S. Lin & W. T. Yang (NMNS); 1♂, same locality, 13.VII.-10.VIII.2004, leg. C. S. Lin & Y. T. Yang (NMNS); 2♂♂, 2♀♀, same locality, 19.VI.2010, leg. C.-F. Lee (TARI); 8♂♂, 9♀♀, Tatachia (塔塔加), 2610 m, 9.VI.2009, leg. C.-F. Lee (TARI); 1♂, same

locality, 20.VII.2009, leg. C.-F. Lee (TARI); 3♂♂, 8♀♀, same locality, 27.IV.2010, leg. C.-F. Lee (TARI); 5♂♂, 4♂♀, same locality, 17.V.2010, leg. C.-F. Lee (TARI); 2♀♀, Tsuifeng (翠峰), 2374 m, 21.VI.1979, leg. K. S. Lin & B. H. Chen (TARI); 8♂♂, 2♀♀, same locality, 3.VI.1980, L. Y. Chou & C. C. Chen (TARI); 2♂♂, 3♀♀, same locality 8.V.1981, leg. K. S. Lin & S. C. Lin (TARI); 42♂♂, 42♀♀, same locality, 25-27.VI.1981, leg. K. S. Lin & W. S. Tang (TARI); 18♂♂, 12♀♀, same locality, 23.V.1982, leg. L. Y. Chou (TARI); 12♂♂, 8♀♀, same locality, IV.1984, leg. K. S. Lin & K. C. Chou (TARI); 6♂♂, same locality, 9.V.1984, leg. K. C. Chou & C. C. Pan (TARI); 1♂, same locality, 23.VII.1984, leg. K. S. Lin (TARI); 3♂♂, 1♀, same locality, 5.VIII.1984, leg. K. S. Lin (TARI); **Taichung**: 1♀, Hsuehshan (雪山), 3886 m, 1.V.2012, leg. T.-H. Lee (TARI); 1♂, 4♀♀, Kukuan (谷關), 730 m, 14-17.X.1980, leg. K. S. Lin & C. H. Wang (TARI); 1♂, Pilu (畢祿), 2584 m, 18.VI.2010, leg. C.-F. Lee (TARI); 1♂, 1♀, Tahsuehshan (大雪山), 2600 m, 7.VI.2010, leg. C.-F. Lee (TARI); **Taitung**: 1♂, Liyuan (栗園), 1793 m, 28.III.2014, leg. J.-C. Chen (TARI); **Taoyuan**: 6♂♂, 5♀♀, Lalashan (拉拉山), 2031 m, 1.IV.2009, leg. C.-F. Lee (TARI); 1♂, 2.IV.2009, leg. H.-J. Chen (TARI); 5♂♂, 13♀♀, same locality, 14.V.2009, leg. C.-F. lee (TARI); 2♀♀, same locality, 4.V.2010, leg. S.-F. Yu (TARI).

Form D (n= 396). **Chiayi**: 3♂♂, 1♀, Alishan (阿里山), 2216 m, 10.V.2011, leg. M.-H. Tsou (TARI); 9♂♂, 7♀♀, same locality, 12.V.2011, leg. C.-F. Lee (TARI); 2♂♂, Tzuchung (自忠), 2280 m, 8.V.2015, leg. J.-C. Chen (TARI); **Ilan**: 1♂, 2♀♀, Ssuyuanyakou (思源啞口), 1948 m, 24.VII.2010, leg. M.-H. Tsou (TARI); 1♀, Taipingshan (太平山), 1950 m, 10.VI.2011, leg. S.-F. Yu (TARI); **Nantou**: 1♀, Hohuanshan (合歡山), 3422 m, 2.VII.2008, leg. M.-H. Tsou (TARI); 2♂♂, 17.V.2009, same locality, leg. M.-H. Tsou (TARI); 2♂♂, same locality, 18.V.2009, leg. C.-F. Lee (TARI); 1♂, Hsiaofengkou (小風口), 3002 m, 9.VIII.2012, leg. C.-F. Lee (TARI); 1♂, 2♀♀, Huakang (華岡), 2575 m, 29.V.2011, leg. M.-H. Tsou (TARI); 1♀, Piliuchi (碧綠溪), 2300 m, 15-31.III.1998, leg. M. M. Yang (NMNS); 4♂♂, 8♀♀, Tatachia (塔塔加), 2610 m, 9.VI.2009, leg. C.-F. Lee (TARI); 2♂♂, 1♀, same locality, 20.VII.2009, leg. C.-F. Lee (TARI); 8♂♂, 1♀, same locality, 27.IV.2010, leg. C.-F. Lee (TARI); 2♂♂, 3♀♀, same locality, 17.V.2010, leg. C.-F. Lee (TARI); 12♂♂, 27♀♀, same locality, 13.V.2015, leg. C.-F. Lee (TARI); 1♂, 1♀, same locality, 1.VII.2015, leg. J.-C. Chen (TARI); 1♂, Tsuifeng (翠峰), 2374 m, 21.VI.1979, leg. K. S. Lin & B. H. Chen (TARI); 2♂♂, 2♀♀, 3.VI.1980, leg. L. Y. Chou & C. C. Chen (TARI); 1♀, same locality, 14-17.X.1980, leg. K. S. Lin & C. H. Wang (TARI); 2♀♀, same locality, 25-27.VI.1981, leg. K. S. Lin & W. S. Tang (TARI); 1♀, IV.1984, leg. K. S. Lin & K. C. Chou (TARI); 1♀, same locality, 5.VIII.1984, leg. K. S. Lin (TARI); 1♀, same locality, 6.VII.2015, leg. C.-F. Lee (TARI); 119♂♂, 113♀♀,

Yuanfeng (鳶峰), 2756 m, 12.VI.2014, leg. C.-F. Lee (TARI); 3♂♂, 5♀♀, same locality, 25.VII.2014, leg. J.-C. Chen (TARI); 1♀, same locality, 29.VII.2014, C.-F. Lee (TARI); **Taichung**: 3♂♂, 1♀, Hsuehshan (雪山), 3886 m, 15.X.2009, leg. W.-B. Yeh (TARI); 3♂♂, 1♀, same locality, 18.VI.2010, leg. W.-B. Yeh (TARI); 1♂, same locality, 3.VIII.2010, leg. W.-B. Yeh (TARI); 1♀, same locality, 9.VI.2011, leg. W.-B. Yeh (TARI); 1♂, 1♀, Pilu (畢祿), 2584 m, 1.VII.2008, leg. S.-F. Yu (TARI); 2♀♀, same locality, 2.VII.2008, leg. M.-H. Tsou (TARI); 1♂, same locality, 17.V.2009, leg. C.-F. Lee (TARI); 2♂♂, 1♀, same locality, 18.VI.2010, leg. C.-F. Lee (TARI); 5♂♂, 7♀♀, same locality, 12.VI.2014, leg. C.-F. Lee (TARI); **Taitung**: 5♂♂, 1♀, Hsiangyangshan (向陽山), 3603 m, 20.VI.2014, leg. J.-C. Chen (TARI); 1♀, Liyuan (栗園), 1793 m, 19.VI.2013, leg. C.-F. Lee (TARI).

Form E (n= 37). **Nantou**: 2♀♀, Huakang (華岡), 2575 m, 29.V.2011, leg. M.-H. Tsou (TARI); 4♂♂, 4♀♀, Tatachia (塔塔加), 2610 m, 9.VI.2009, leg. C.-F. Lee (TARI); 1♂, 2♀♀, same locality, 20.VII.2009, leg. C.-F. Lee (TARI); 1♀, same locality, 21.VII.2009, leg. S.-F. Yu (TARI); **Taichung**: 1♂, Hsuehshan (雪山), 3886 m, 8.VI.2008, leg. W.-B. Yeh (TARI); 5♂♂, 2♀♀, same locality, 17-18.VI.2010, leg. W.-B. Yeh (TARI); 1♂, same locality, 3.VIII.2010, leg. W.-B. Yeh (TARI); 5♂♂, 1♀, same locality, 9.VI.2011, leg. W.-B. Yeh (TARI); 3♂♂, 5♀♀, same locality, 29.IV.-28.VI.2012, leg. L.-P. Hsu (TARI).

Form F (n= 27). **Chiayi**: 1♀, Alishan (阿里山), 2216 m, 27.VI.2010, leg. U. Ong (TARI); **Hualien**: 1♀, Pilu (畢祿), 2584 m, 17.V.2009, leg. C.-F. Lee (TARI); 1♀, Tayuling (大禹嶺), 2560 m, 9-16.VI.1980, leg. K. S. Lin & B. H. Chen (TARI); **Nantou**: 3♀♀, Huakang (華岡), 2575 m, 29.V.2011, leg. M.-H. Tsou (TARI); 2♂♂, Tatachia (塔塔加), 2610 m, 9.VI.2009, leg. C.-F. Lee (TARI); 3♀♀, same locality, 20.VII.2009, leg. C.-F. Lee (TARI); 7♂♂, 5♀♀, Yuanfeng (鳶峰), 2756 m, 12.VI.2014, leg. C.-F. Lee (TARI); **Taichung**: 1♀, Pilu (畢祿), 2584 m, 1.VII.2008, leg. S.-F. Yu (TARI); 3♀♀, same locality, 17.V.2009, leg. M.-H. Tsou (TARI); 18.VI.2010, leg. C.-F. Lee (TARI).
