# Supplementary material for: Revision of Mandarella Duvivier from Taiwan, with a new species, new synonymies and identities of highly variable species (Insecta, Chrysomelidae, Galerucinae, Alticini)
Source: Zookeys. 2016 Feb 23;(568):23–49. doi: 10.3897/zookeys.568.7125 (PMC4829668; doi:10.3897/zookeys.568.7125)
Supplement: Supplementary material 3 — Mandarella flaviventrites, other material examined [file zookeys-568-023-s003.pdf]

Other Material examined. Forms G and H (n= 582). **Chiayi**: 1♀, Tzuchung (自忠), 2280 m, 21.IX.2009, leg. M.-H. Tsou (TARI); 2♂♂, Alishan (阿里山), 2216 m, 5-9.VIII.1981, leg. L. Y. Chou & S. C. Lin (TARI); 1♂, same locality, 17-20.VIII.1982, leg. K. C. Chou & C. C. Pan (TARI); 1♂, 2♀♀, same locality, 25.IV.2009, leg. H.-J. Chen (TARI); 1♂, same locality, 27.VI.2010, leg. U. Ong (TARI); 1♂, 3♀♀, same locality, 12.V.2011, leg. C.-F. Lee (TARI); 5♂♂, same locality, 22.IX.2011, leg. C.-F. Lee (TARI); **Hsinchu**: 3♂♂, 3♀♀, Kuanwu (觀霧), 2000 m, 30.IV.2010, leg. M.-H. Tsou (TARI); 19♂♂, 3♀♀, Lupi (魯壁), 1500 m, 4.IV.2009, leg. M.-H. Tsou (TARI); 6♂♂, 3♀♀, same locality, 18.IV.2009, leg. M.-H. Tsou (TARI); 4♂♂, 3♀♀, same locality, 12.IV.2015, leg. M.-H. Tsou (TARI); 1♀, Talu trail (大鹿林道), 2000 m, 27.IV.2008, leg. Y.-L. Lin (TARI); **Hualien**: 1♀, Piliu (碧綠), 2150 m, 17.V.2009, leg. C.-F. Lee (TARI); 2♀♀, 31.V.2011, leg. M.-H. Tsou (TARI); 3♀♀, Tayuling (大禹嶺), 2560 m, 9-16.VI.1980, leg. K. S. Lin & B. H. Chen (TARI); **Ilan**: 1♂, 1♀, Taipingshan (太平山), 1950 m, 26-28.VII.1983, leg. L. Y. Chou (TARI); 1♀, same locality, 3.VI.2007, leg. S.-F. Yu (TARI); 1♀, same locality, 5.V.2009, leg. J.-C. Chen (TARI); 1♀, same locality, 30.V.2008, H.-J. Chen (TARI); 1♂, 6♀♀, Ssuyuanyakou (思源啞口), 1948 m, 25.IV.2009, leg. C.-F. Lee (TARI); 2♂♂, 13♀♀, same locality, 28.IV.2009, leg. M.-H. Tsou (TARI); 1♂, same locality, 9.VI.2009, leg. S.-F. Yu (TARI); 1♀, same locality, 24.VII.2010, leg. M.-H. Tsou (TARI); 1♀, same locality, 6.V.2011, leg. S.-F. Yu (TARI); 1♂, 1♀, Yuanyanghu (鴛鴦湖), 1670 m, 19.VIII.2010, leg. H.-H. Lee (TARI); 7♂♂, 3♀♀, same locality, 22.VIII.2011, leg. C.-F. Lee (TARI); **Kaohsiung**: 1♀, Chungchihkuan (中之關), 1930 m, 15.IV.2012, leg. L.-P. Hsu (TARI); 1♀, same locality, 17.IV.2012, leg. L.-P. Hsu (TARI); 1♂, 1♀, same locality, 10-13.X.2012, leg. L.-P. Hsu (TARI); 1♂, Tengchih (藤枝), 1550 m, 18.II.2007, leg. S.-F. Yu (TARI); 7♂♂, 1♀, Tianchi (天池), 2280 m, 11.X.2012, leg. L.-P. Hsu (TARI); 1♀, Tona (多納), 500 m, 25.II.2013, leg. Y.-T. Chung (TARI); **Nantou**: 1♂, 1♀, Chingching (清境), 1750 m, 5.III.2007, leg. H.-J. Chen (TARI); 1♂, 1♀, Fenghuangshan (鳳凰山), 1700 m, 10.V.2010, leg. Y.-T. Wang (TARI); 3♀♀, 10.VIII.2011, leg. M.-H. Tsou (TARI); 1♂, 1♀, Hohuanshan (合歡山), 3422 m, 17-18.V.2009, leg. C.-F. Lee (TARI); 16♂♂, 12♀♀, Hsitou (溪頭), 1150 m, 6.V.2009, leg. C.-F. Lee (TARI); 4♀♀, same locality, 15.VI.2011, leg. C.-F. Lee (TARI); 1♀, same locality, 10.VIII.2011, leg. C.-F. Lee (TARI); 4♂♂, 2♀♀, Huakang (華岡), 2575 m, 12.IX.2010, leg. C.-F. Lee (TARI); 1♀, same locality, 30.V.2011, leg. M.-H. Tsou (TARI); 1♀, Meifeng (梅峰), 2100 m, 10.V.1979, leg. K. C. Chou (TARI); 1♀, same locality, 2-4.VI.1980, L. Y. Chou & C. C. Chen (TARI); 1♀, same locality, 6-8.VI.1980, leg. K. S. Lin & B. H. Chen (TARI); 3♂♂, 2♀♀, same locality, 26.VIII.1980, leg. K. S. Lin & C. H. Wang (TARI); 2♀♀, same locality, 5-9.X.1980, leg. C. C. Chen & C. C. Chien (TARI); 2♂♂, 9♀♀, same locality, 7-9.V.1981, leg. K.

S. Lin & S. C. Lin (TARI); 2♀♀, same locality, 24-26.VI.1981, leg. K. S. Lin & W. S. Tang (TARI); 37♂♂, 21♀♀, same locality, 28-29.VIII.1981, leg. L. Y. Chou & S. C. Lin (TARI); 28♂♂, 5♀♀, same locality, 31.VIII.-2.IX.1982, leg. L. Y. Chou & K. C. Chou (TARI); 1♂, 5♀♀, same locality, 4-7.X.1982, leg. K. C. Chou (TARI); 2♀♀, same locality, 19-21.IV.1983, leg. K. C. Chou & S. P. Huang (TARI); 1♂, same locality, 22.VII.1992, leg. C. S. Lin (NMNS); 1♀, same locality, 13.VI.-18.VII.2001, leg. C. S. Lin & W. T. Yang (NMNS); 1♂, same locality, 13.VIII.-11.IX.2002, leg. C. S. Lin & W. T. Yang (NMNS); 2♀♀, same locality, 11.V.-13.VII.2004, leg. C. S. Lin & W. T. Yang (NMNS); 1♀, same locality, 3.VII.2008, leg. M.-H. Tsou (TARI); 2♀♀, same locality, 14-15.IX.2009, leg. S.-F. Yu (TARI); 6♀♀, Piliuchi (碧綠溪), 2300 m, 15-30.V.1997, leg. M. M. Yang (NMNS); 1♀, Shanlinhsi (杉林溪), 1600 m, 26.IV.2014, leg. J.-C. Chen (TARI); 2♂♂, Sungkang (松岡), 2000 m, 15-17.VIII.1984, leg. K. C. Chou (TARI); 4♂♂, 6♀♀, same locality, 13-15.IX.1984, leg. K. S. Lin & S. L. Lin (TARI); 1♂, 1♀, Tatachia (塔塔加), 2610 m, 20.VII.2009, leg. C.-F. Lee (TARI); 2♀♀, same locality, 2610 m, 21.VII.2009, leg. S.-F. Yu (TARI); 1♀, same locality, 30.X.2009, leg. C.-F. Lee (TARI); 5♂, 6♀♀, same locality, 27.IV.2010, leg. C.-F. Lee (TARI); 1♀, same locality, 17.V.2010, leg. C.-F. Lee (TARI); 1♂, same locality, 13.V.2015, leg. C.-F. Lee (TARI); 1♀, Tsuifeng (翠峰), 2374 m, 21.VI.1979, leg. K. S. Lin & B. H. Chen (TARI); 1♀, 25-27.VI.1981, leg. K. S. Lin & W. S. Tang (TARI); 3♂♂, 1♀, same locality, 1-3.VIII.1981, leg. T. Lin & W. S. Tang (TARI); 1♂, 2♀♀, same locality, 27.VIII.1981, leg. L. Y. Chou & S. C. Lin (TARI); 3♀♀, same locality, 23.V.1982, leg. L. Y. Chou (TARI); 1♂, 1♀, same locality, 20.IV.1983, leg. K. C. Chou & S. P. Huang (TARI); 1♀, same locality, IV.1984, leg. K. S. Lin & K. C. Chou (TARI); 2♀♀, same locality, 9.V.1984, leg. K. C. Chou & C. C. Pan (TARI); 3♂♂, same locality, 12-14.IX.1984, leg. K. S. Lin & S. C. Lin (TARI); 3♀♀, same locality, 11.VI.2014, leg. C.-F. Lee (TARI); 3♀♀, Tungfu (同富), 1500 m, 8.V.2011, leg. C.-F. Lee (TARI); 1♀, Tungpu (東埔), 1200 m, 25-29.IX.1980, leg. L. Y. Chou & T. Lin (TARI); 8♀♀, same locality, 5-8.X.1981, leg. T. Lin & W. S. Tang (TARI); 11♀♀, same locality, 1120 m, 18-23.XI.1981, leg. T. Lin & W. S. Tang (TARI); 2♂♂, 1♀, Tunyuan (屯原), 1900 m, 14.V.2013, leg. B.-X. Guo (TARI); 1♀, same locality, 24.V.2014, leg. J.-C. Chen (TARI); 3♀♀, Wushe (霧社), 1148 m, 6-11.V.1981, leg. K. S. Lin & S. C. Lin (TARI); 1♂, same locality, 30.VIII.-2.IX.1982, leg. L. Y. Chou & K. C. Chou (TARI); 2♀♀, same locality, 7-8.X.1982, leg. K. C. Chou (TARI); 2♂♂, 1♀, Yuanfeng (鳶峰), 2756 m, 12.VI.2014, leg. C.-F. Lee (TARI); 1♂, Yushih (幼獅), 2000 m, 4.VIII.1981, leg. T. Lin & W. S. Tang (TARI); **Pingtung**: 7♂♂, 3♀♀, Jinshuiying (浸水營), 1450 m, 5.IV.2011, leg. J.-C. Chen (TARI); 3♂♂, same locality, 14.IV.2011, leg. J.-C. Chen (TARI); 1♂, same locality, 21.IV.2011, leg. J.-C. Chen (TARI); 1♂, 1♀, 3.V.2011, leg. J.-C. Chen

(TARI); 2♂♂, 10♀♀, same locality, 12.IV.2012, leg. C.-F. Lee (TARI); 1♀, same locality, 28.IV.2012, leg. M.-H. Tsou (TARI); 2♀♀, same locality, 4.IV.2013, leg. J.-C. Chen (TARI); 1♂, same locality, 6.IV.2013, leg. W.-C. Liao (TARI); 4♂♂, 8♀♀, same locality, 9.IV.2014, leg. J.-C. Chen (TARI); 1♂, 5♀♀, same locality, 27.IV.2014, leg. W.-C. Liao (TARI); 1♂, same locality, 30.IV.2014, leg. Y.-T. Chung (TARI); 33♂♂, 13♀♀, Tahanshan (大漢山), 1200 m, 2.IV.2015, leg. Y.-T. Chung (TARI); **Taichung**: 1♀, Chiapaotai (佳保台), 750 m, 14-18.X.1980, leg. K. S. Lin & C. H. Wang (TARI); 2♀♀, Kukuan (谷關), 730 m, 14-17.X.1980, leg. K. S. Lin & C. H. Wang (TARI); 1♀, Tahsuehshan (大雪山), 2600 m, 22.IX.2007, leg. M.-H. Tsou (TARI); 3♂♂, same locality, 21.IV.2010, leg. C.-F. Lee (TARI); 1♀, same locality, 7.VI.2010, leg. C.-F. Lee (TARI); 1♂, 22.III.2011, leg. C.-F. Lee (TARI); 1♂, same locality, 19.IV.2011, leg. J.-C. Chen (TARI); 1♂, same locality, 23.VII.2011, leg. J.-C. Chen (TARI); 1♀, Wuling (武陵), 1900 m, 27-29.VI.1979, leg. K. S. Lin & L. Y. Chou (TARI); 1♂, Yuantsuishan (鳶嘴山), 2130 m, 16.VII.2010, leg. J.-C. Chen (TARI); **Taitung**: 2♂♂, 1♀, Hsiangyang (向陽), 2320 m, 23.VI.2010, leg. M.-H. Tsou (TARI); 1♀, same locality, 8.VII.2010, leg. J.-C. Chen (TARI); 1♂, same locality, 1.IV.2011, leg. J.-C. Chen (TARI); 1♂, same locality, 5.IV.2012, leg. J.-C. Chen (TARI); 2♀♀, same locality, 14.VIII.2012, leg. C.-F. Lee (TARI); 1♀, same locality, 9.V.2013, leg. J.-C. Chen (TARI); 1♀, same locality, 19.IV.2014, leg. J.-C. Chen (TARI); 1♂, 1♀, same locality, 17.V.2014, leg. J.-C. Chen (TARI); 1♀, Litao (利稻), 1068 m, 29.III.2011, leg. M.-H. Tsou (TARI); 1♀, Liyuan (栗園), 1793 m, 23.VI.2010, leg. M.-H. Tsou (TARI); 6♂♂, 4♀♀, same locality, 29.III.2011, leg. C.-F. Lee (TARI); 2♂♂, same locality, 19.VI.2013, leg. C.-F. Lee (TARI); 1♀, same locality, 19.VI.2013, leg. B.-X. Guo (TARI); 1♀, same locality, 24.VII.2013, leg. C.-F. Lee (TARI); 3♂♂, same locality, 24.I.2014, leg. W.-C. Huang (TARI); 1♂, 1♀, same locality, 24.II.2014, leg. J.-C. Chen (TARI); 1♂, same locality, 14.III.2014, leg. W.-C. Huang (TARI); 1♂, 1♀, same locality, 28.III.2014, leg. J.-C. Chen (TARI); 1♀, same locality, 19.IV.2014, leg. W.-C. Huang (TARI); 1♂, Motien (摩天), 1546 m, 23.VI.2010, leg. M.-H. Tsou (TARI); 1♂, same locality, 14.IX.2010, leg. C.-F. Lee (TARI); 4♂♂, 6♀♀, same locality, 5.X.2010, leg. C.-F. Lee (TARI); 1♂, same locality, 23.V.2011, leg. C.-F. Lee (TARI); 3♂♂, 1♀, same locality, 19.VI.2011, leg. C.-F. Lee (TARI); 4♂♂, 2♀♀, Wulu (霧鹿), 780 m, 29.III.2011, leg. M.-H. Tsou (TARI); **Taoyuan**: 1♀, Hsuehwunao (雪霧鬧), 1450 m, 3.IV.2011, leg. M.-H. Tsou (TARI); 3♂♂, 1♀, Lalashan (拉拉山), 2031 m, 1.IV.2009, leg. C.-F. Lee (TARI); 1♂, same locality, 2.V.2009, leg. M.-H. Tsou (TARI); 1♀, same locality, 14.V.2009, leg. C.-F. Lee (TARI); 1♀, same locality, 23.VII.2009, leg. H. Lee (TARI); 1♂, same locality, 4.V.2010, leg. S.-F. Yu (TARI); 1♂, Tamanshan (塔曼山), 2130 m, 2.VIII.2008, leg. S.-F. Yu (TARI).

Form I (n= 135). **Chiayi**: 1♂, 2♀♀, Alishan (阿里山), 2216 m, 22.IX.2011, leg. C.-F. Lee (TARI); **Hualien**: 1♀, Piliu (碧綠), 2150 m, 25.IX.2010, leg. W.-P. Chan (TARI); 1♂, Tayuling (大禹嶺), 2560 m, 12-15.IX.1980, leg. K. S. Lin & C. H. Wang (TARI); 4♂♂, 1♀, same locality, 3.IX.2008, leg. H. H. Lin (NMNS); **Ilan**: 4♂♂, 2♀♀, Nanhutashan (南湖大山), 3740 m, 11.VIII.2014, leg. J.-C. Chen (TARI); 2♂♂, 1♀, Ssuyuanyakou (思源啞口), 1948 m, 31.VII.2009, leg. H.-J. Chen (TARI); 3♂♂, 2♀♀, Yuanyanghu (鴛鴦湖), 1670 m, 22.VIII.2011, leg. C.-F. Lee (TARI); **Kaoshiung**: 1♂, Chungchihkuan (中之關), 1930 m, 10-13.X.2012, leg. L.-P. Hsu (TARI); 1♂, Kueiku (檜谷), 2450 m, 14.IV.2012, leg. L.-P. Hsu (TARI); 1♀, Tengchih (藤枝), 1550 m, 8.V.2013, leg. W.-C. Liao (TARI); 1♂, 1♀, Tianchi (天池), 2280 m, 11.X.2012, leg. L.-P. Hsu (TARI); **Nantou**: 3♂♂, Hohuanshan (合歡山), 3422 m, 26.VII.2010, leg. M.-H. Tsou (TARI); 2♂♂, 1♀♀, Hsiaofengkou (小風口), 3002 m, 9.VIII.2012, leg. C.-F. Lee (TARI); 2♂♂, 4♀♀, Huakang (華岡), 2575 m, 12.IX.2010, leg. C.-F. Lee (TARI); 1♀, Tatachia (塔塔加), 2610 m, 20.VII.2009, leg. C.-F. Lee (TARI); 10♂♂, 25♀♀, same locality, 21.IX.2009, leg. C.-F. Lee (TARI); 8♀♀, same locality, 30.X.2009, leg. C.-F. Lee (TARI); 2♀♀, same locality, 17.XI.2009, leg. C.-F. Lee (TARI); 2♂♂, Tsuifeng (翠峰), 2374 m, 27.VIII.1981, leg. L. Y. Chou & S. C. Lin (TARI); 3♂♂, same locality, 1-3.IX.1982, leg. L. Y. Chou & K. C. Chou (TARI); 1♂, same locality, 15-16.VIII.1984, leg. K. C. Chou (TARI); 2♂♂, same locality, 12-14.IX.1984, leg. K. S. Lin & S. C. Lin (TARI); 9♂♂, 2♀♀, Yuanfeng (鳶峰), 2756 m, 9-19.V.1998, leg. C. S. Lin & W. T. Yang (NMNS); **Taichung**: 2♂♂, Hsuehshan (雪山), 3886 m, 7.VIII.2008, leg. W. T. Yang (NMNS); 1♀, same locality, 14.X.2009, leg. W.-B. Yeh (TARI); 1♀, same locality, 3.VIII.2010, leg. W.-B. Yeh (TARI); 1♀, same locality, 30.IX.2010, leg. W.-B. Yeh (TARI); 2♀♀, same locality, 2.X.2010, leg. W.-B. Yeh (TARI); 2♀♀, 7.X.2011, leg. W.-B. Yeh (TARI); 1♂, 4♀♀, same locality, 2.IX.2014, leg. J.-C. Chen (TARI); 1♂, Tahsuehshan (大雪山), 2600 m, 19-20.VIII.2008, leg. Liang, Chen & Fu (NMNS); 4♀♀, same locality, 18.X.2008, leg. H. Lee (TARI); 6♂♂, Wuling (武陵), 1900 m, 11.VIII.2014, leg. H. Lee (TARI); **Taitung**: 1♂, 2♀♀, Motien (摩天), 1546 m, 5.X.2010, leg. C.-F. Lee (TARI); 1♀, Hsiangyang (向陽), 2320 m, 27.IX.2007, leg. J.-F. Tsai (TARI); 1♀, same locality, 12.VII.2012, leg. J.-C. Chen (TARI); 10♂♂, same locality, 14.VIII.2012, leg. C.-F. Lee (TARI).
